# Comparative Performance and Species-Specific Recovery Biases of Culture-Based Methods for *Campylobacter* Detection in Food Products: A Systematic Review and Meta-Analysis

**DOI:** 10.3390/vetsci13050415

**Published:** 2026-04-23

**Authors:** Chatruthai Meethai, Preeda Phothawon, Janet Yakubu Nale, Sueptrakool Wisessombat

**Affiliations:** 1 Department of Medical Technology, School of Allied Health Sciences, Walailak University, Nakhon Si Thammarat 80160, Thailand; chatruthai.me@wu.ac.th (C.M.); preeda.ph@wu.ac.th (P.P.); 2 Center of Excellence Research for Melioidosis and Microorganisms (CERMM), Walailak University, Nakhon Si Thammarat 80160, Thailand; 3 Centre for Epidemiology and Planetary Health, School of Veterinary Medicine and Biosciences, Scotland’s Rural College, Inverness IV2 5NA, UK; janet.nale@sruc.ac.uk

**Keywords:** *Campylobacter*, *C. jejuni*, diagnostic accuracy, Bolton broth, direct culture, species-specific bias, polymyxin B, food safety, meta-analysis, systematic review

## Abstract

*Campylobacter* is the primary bacterial cause of foodborne gastroenteritis globally. While international standards recommend a tiered approach for detection, emerging evidence suggests that selective protocols may introduce species-specific recovery biases. This systematic review and meta-analysis evaluated the diagnostic performance of established culture-based detection protocols across diverse food matrices. Following PRISMA 2020 guidelines, we searched multiple databases for studies reporting 2 × 2 diagnostic accuracy data through October 2024. Ten studies comprising 43 method comparisons and 4599 samples met the inclusion criteria. The overall pooled sensitivity was 95.8% (95% CI: 93.6–97.4%), and the specificity was 90.2% (95% CI: 86.8–92.9%). Even with a limited number of comparisons (*n* = 2), direct culture demonstrated high diagnostic sensitivity (99.1%) and significantly faster turnaround times. Crucially, selective enrichment exhibited a profound species-specific bias: *C. jejuni* showed 59.4 percent lower recovery than *C. coli* in Bolton broth, likely due to differential polymyxin B susceptibility. These findings highlight the importance of context-dependent method selection within the ISO 10272-1:2017 framework, suggesting that direct culture (Procedure C) should be prioritized for high-contamination matrices to ensure unbiased recovery of *C. jejuni*. Large-scale multicenter validation is warranted to confirm these exploratory findings for direct culture.

## Simple Summary

*Campylobacter* is the leading cause of bacterial food poisoning worldwide, often linked to contaminated poultry. To protect public health, food safety authorities rely on accurate laboratory tests defined by international standards (ISO 10272-1:2017), which use a tiered approach involving selective enrichment (Procedures A and B) and direct plating (Procedure C). Our systematic review and meta-analysis of 4599 samples identify a critical biological factor influencing these methods. We found that the selective enrichment process, which utilizes specific antibiotics such as polymyxin B, systematically suppresses the growth of *C. jejuni*—the species responsible for nearly 90% of human infections. In contrast, direct culture (Procedure C) demonstrated high sensitivity (99.1%) and provided results in half the time (48 h vs. 96 h) for high-contamination matrices. These findings provide a biological rationale for the situational prioritization of direct culture for fresh poultry products, thereby ensuring more reliable food safety surveillance and consumer protection.

## 1. Introduction

*Campylobacter* spp., particularly *C. jejuni* and *C. coli*, are the most frequently reported bacterial causes of foodborne gastroenteritis worldwide, with an estimated 96 million cases annually [1,2]. In the European Union, *Campylobacter* infections have consistently exceeded all other bacterial foodborne pathogens since 2005, accounting for approximately 246,000 confirmed cases in 2023 alone [3]. In the United States, the Centers for Disease Control and Prevention (CDC) estimates 1.5 million domestically acquired *Campylobacter* infections occur each year, resulting in substantial morbidity and healthcare costs [4].

Poultry products, particularly chicken meat and carcasses, serve as primary reservoirs and vehicles for transmission of human campylobacteriosis, with contamination rates ranging from 20% to 100%, depending on geographic region and production practices [5,6]. Other important food sources include raw milk, pork, beef, and fresh produce contaminated through environmental pathways [7,8]. The low infectious dose and widespread contamination of retail poultry underscore the critical importance of accurate detection methods for food safety surveillance, outbreak investigation, and risk assessment [9,10].

Accurate detection and enumeration of *Campylobacter* in food products serve multiple critical functions in public health protection. First, routine surveillance of food products at retail and production facilities enables the identification of contamination trends, the assessment of intervention effectiveness, and the early warning of potential outbreaks [11,12]. Second, during outbreak investigations, rapid and reliable detection methods are essential for source attribution and implementation of control measures [13]. Third, quantitative data on *Campylobacter* levels in food products inform quantitative microbial risk assessment (QMRA) models that guide regulatory standards and industry best practices [14,15].

However, *Campylobacter*’s fastidious nature poses unique challenges for detection. These organisms are microaerophilic, requiring reduced oxygen tension (3–15% O_2_) for growth, and are thermophilic, with optimal growth at 37–42 °C [16]. Additionally, *Campylobacter* readily enters a viable but non-culturable (VBNC) state under stress conditions commonly encountered during food processing and storage, including exposure to atmospheric oxygen, temperature fluctuations, and osmotic stress [17,18]. VBNC cells retain metabolic activity and pathogenic potential but fail to form colonies on standard culture media, leading to systematic underestimation of contamination levels [19,20].

Despite advances in molecular detection technologies, culture-based methods remain the gold standard for *Campylobacter* detection in food products and are mandated by international standards, including ISO 10272-1:2017 [21]. Culture methods offer several advantages: they confirm organism viability, enable strain isolation for further characterization (serotyping, antimicrobial susceptibility testing, whole-genome sequencing), allow quantitative enumeration, and provide definitive evidence for regulatory enforcement actions [22,23]. The general workflow involves selective enrichment in specialized broths, plating on selective agar plates, and confirmation by biochemical and molecular tests [24].

The ISO 10272-1:2017 standard specifies a two-stage enrichment protocol: initial incubation at 37 °C for 4–6 h to resuscitate stressed cells, followed by selective enrichment at 41.5 °C for 40–48 h under microaerobic conditions [21]. After enrichment, samples are streaked onto selective agars and incubated at 41.5 °C for 24–48 h. Presumptive colonies are confirmed through Gram staining, oxidase testing, and species-specific PCR [25]. While this standardized approach provides a framework for detection, substantial variation exists in enrichment broth formulations, selective agar compositions, and confirmation protocols across laboratories and studies [26,27].

Three primary enrichment broth formulations dominate *Campylobacter* detection protocols, each with distinct selective agents and growth supplements: Bolton Broth, developed in the 1980s, contains a nutrient broth base supplemented with lysed horse blood (5%), sodium metabisulfite, sodium pyruvate, and selective antimicrobial agents [28,29]. Bolton broth is widely used in Europe and North America and is specified in ISO 10272-1:2017 as a primary enrichment option [21].

Preston Broth contains a nutrient broth base with selective antimicrobials and requires supplementation with FBP supplement to achieve optimal performance [30]. However, recent evidence indicates that Preston broth is frequently used without FBP supplementation, resulting in dramatically reduced sensitivity, particularly for *C. coli* and *C. lari* [31]. When properly supplemented, Preston broth performs comparably to Bolton broth, but the additional preparation step and the potential for omission pose practical limitations [32].

Modified Charcoal Cefoperazone Deoxycholate Agar (mCCDA) serves as both a selective plating medium and, in some protocols, a direct plating medium without prior enrichment [33]. The charcoal component neutralizes toxic oxygen metabolites, while cefoperazone and deoxycholate provide selectivity against Gram-positive bacteria and most Gram-negative competitors [34]. Direct plating on mCCDA enables quantitative enumeration but suffers from reduced sensitivity at low contamination levels and interference from extended-spectrum beta-lactamase (ESBL)-producing *Escherichia coli*, which has emerged as a significant problem in recent years [35,36].

Despite the central role of culture-based methods in *Campylobacter* detection, several critical knowledge gaps persist. First, the diagnostic accuracy (sensitivity and specificity) of different enrichment broth protocols across diverse food matrices remains incompletely characterized, with individual studies reporting widely varying performance metrics [37,38]. Second, the impact of contamination level on detection sensitivity has not been systematically quantified, despite recognition that low-level contamination poses particular challenges [39]. Third, comparative performance data for Bolton versus Preston broth are limited and confounded by inconsistent use of FBP supplementation [31]. Fourth, emerging challenges, including VBNC cells and ESBL *E. coli* interference, have been documented in individual studies but not synthesized across the literature [35,40].

The primary objective of this systematic review and meta-analysis was to synthesize evidence on the diagnostic accuracy of culture-dependent methods using enrichment broths for detecting *Campylobacter* spp. in food products.

## 2. Materials and Methods

### 2.1. Protocol and Registration

This study was conducted according to the Preferred Reporting Items for Systematic Reviews and Meta-Analyses guidelines [41] and the Cochrane Handbook for Systematic Reviews of Diagnostic Test Accuracy [42]. The PRISMA Checklist was followed for each section of the meta-analysis (https://www.prisma-statement.org/). The protocol was prospectively designed and registered in PROSPERO (International Prospective Register of Systematic Reviews) on 6 December 2024, under registration number CRD1179196.

The protocol specified the population (food products tested for *Campylobacter* contamination), index tests (culture-based detection methods including enrichment broths and direct culture), reference standards (validated culture-based or molecular methods), outcomes (diagnostic accuracy measures including sensitivity and specificity), and study designs (cross-sectional, cohort, and experimental studies reporting diagnostic test accuracy). No deviations from the registered protocol occurred during the review process.

### 2.2. Study Eligibility and the PIRD Framework

Studies were selected using the Population, Index test, Reference standard, and Design (PIRD) framework. Recommended for diagnostic test accuracy reviews [43].

Population (P): Studies evaluating fresh or frozen food products intended for human consumption, including but not limited to raw poultry (chicken, turkey), red meat (beef, pork), dairy products (raw milk), vegetables, and seafood. Studies were included regardless of contamination type (natural or artificial inoculation) or contamination level. Clinical specimens, environmental samples not directly related to food products, and animal feed samples were excluded.

Index Test (I): Culture-based detection methods for *Campylobacter* species, including: (1) enrichment in selective liquid media (Bolton broth, Preston broth, *Campylobacter* Enrichment Broth [CEB], or modified variants) followed by plating on selective agar, (2) direct plating on selective agar without enrichment (modified Charcoal Cefoperazone Deoxycholate Agar [mCCDA], *Campylobacter* Blood-Free Selective Agar [CCDA], or similar), or (3) combined approaches using pre-enrichment in non-selective media followed by selective enrichment. Studies using exclusively molecular methods (PCR, loop-mediated isothermal amplification [LAMP], whole-genome sequencing) without culture-based confirmation were excluded.

Reference Standard (R): A validated reference standard capable of correctly classifying the presence or absence of *Campylobacter* in food samples. Acceptable reference standards included: (1) composite culture-based methods combining multiple enrichment broths and/or direct culture, (2) culture methods with molecular confirmation (PCR, real-time PCR, or DNA sequencing for species identification), (3) validated protocols conforming to international standards (ISO 10272-1:2017), or (4) any combination of the above with demonstrated validity. Studies that used the same method for both the index test and the reference standard or used undefined or poorly described reference standards were excluded.

Design (D): Primary research studies reporting original data on diagnostic test accuracy, including cross-sectional studies, prospective or retrospective cohort studies, and experimental validation studies. Studies must have provided sufficient data to construct 2 × 2 contingency tables (true positives [TP], false positives [FP], true negatives [TN], and false negatives [FN]) or to calculate sensitivity and specificity with 95% confidence intervals. Systematic reviews, meta-analyses, narrative reviews, editorials, commentaries, letters, conference abstracts without full data, and studies reporting only prevalence data without diagnostic accuracy metrics were excluded.

Additional Criteria: Studies published in the English language from 1 January 2000, to 15 January 2026, were eligible. The year 2000 was selected as the start date to focus on modern detection methods and to align with the publication of key methodological standards (ISO 10272:2006 [21]). Studies published in languages other than English were excluded due to resource constraints and the predominance of food microbiology literature in English-language journals.

### 2.3. Search Strategy

A comprehensive systematic search was conducted across five electronic databases and supplemented by hand-searching of reference lists and forward citation tracking. The search was performed through October 2024 with no language restrictions applied to the initial search.

Electronic Databases Searched:

PubMed/MEDLINE (National Library of Medicine): The search strategy combined Medical Subject Headings (MeSH) terms and text words using Boolean operators. The full search string was: (“*Campylobacter*”[Mesh] OR Campylobacter*[tiab]) AND (“Food Microbiology”[Mesh] OR “Food Contamination”[Mesh] OR food*[tiab] OR poultry[tiab] OR chicken*[tiab] OR meat[tiab] OR retail[tiab]) AND (“Sensitivity and Specificity”[Mesh] OR “Diagnostic Test”[tiab] OR detection[tiab] OR isolation[tiab] OR enumeration[tiab] OR culture[tiab] OR “diagnostic accuracy”[tiab]) AND (Bolton[tiab] OR Preston[tiab] OR enrichment[tiab] OR “selective medium”[tiab] OR mCCDA[tiab] OR “direct culture”[tiab] OR “direct plating”[tiab]) AND English[lang]. Filters applied: publication date (2000–2026), English language, article type (excluding reviews, editorials, letters, comments). This search yielded 87 records.

The complete search strategies for all databases are detailed in the Supplementary Materials (Section S1 and Table S1).

### 2.4. Study Selection

Studies were selected using the Population, Index test, Reference standard, and Design (PIRD) framework. To address the conditional nature of *Campylobacter* detection as defined by ISO 10272-1:2017, the framework was applied to evaluate performance across a spectrum of food matrices and contamination states. Specifically, we included studies investigating high-contamination matrices (e.g., fresh poultry and caecal contents), where direct plating (Procedure C) is often used, alongside low-contamination or processed products, where selective enrichment (Procedures A and B) is mandated.

To ensure high methodological comparability and minimize incorporation bias, we included only studies that used a validated reference standard capable of definitive classification, such as the full ISO 10272-1:2017 protocol or culture-based methods supplemented by molecular confirmation (PCR/MALDI-TOF). Furthermore, we specifically audited each study for critical technical parameters, including incubation conditions (temperature and duration), selective plating media, and confirmatory methods, ensuring that the extracted 2 × 2 data reflected equivalent microbiological challenges across diverse reports.

The study selection process was designed to ensure high methodological comparability and to minimize incorporation bias. Beyond initial screening, full-text articles were rigorously audited for the quality of their reference standards. We included only those studies that used a validated reference standard capable of definitive classification, such as the full ISO 10272-1:2017 protocol or culture-based methods supplemented by molecular confirmation (PCR/MALDI-TOF). Studies relying on self-referencing index tests or poorly defined standards were excluded to maintain analytical rigor. Furthermore, to ensure the comparability of findings across diverse reports, we prioritized studies with standardized processing conditions for food matrices (e.g., specific poultry meat or carcass rinse), thereby ensuring that the extracted 2 × 2 data reflected equivalent microbiological challenges. Detailed reasons for exclusion at the full-text stage are documented in Supplementary Materials (Section S2 and Table S2).

### 2.5. Data Extraction

Data extraction was performed independently by two reviewers using a standardized and piloted Excel-based form. Extracted information included study characteristics, food matrix details, microbiological protocols, and 2 × 2 diagnostic accuracy data. Discrepancies were resolved through consensus or by a third-party adjudicator. Detailed reliability statistics (Cohen’s kappa and ICC) and the full data extraction protocol are provided in Supplementary Table S3.

### 2.6. Quality Assessment

Methodological quality and risk of bias were evaluated using the QUADAS-2 tool [44] across four domains: patient selection, index test, reference standard, and flow and timing. Assessments were conducted independently by two reviewers, and inter-rater agreement was assessed using Cohen’s kappa (κ = 0.88). The results were used to inform sensitivity analyses and contextualize the strength of evidence. Full assessment criteria and signaling questions are detailed in Supplementary Sections S4 and S5.

### 2.7. Data Synthesis and Statistical Analysis

#### 2.7.1. Diagnostic Accuracy

For each study or method comparison, sensitivity and specificity were calculated from 2 × 2 contingency tables using standard formulas: sensitivity = TP/(TP + FN) and specificity = TN/(TN + FP). When studies reported sensitivity and specificity directly without providing 2 × 2 tables, we back-calculated TP, FP, TN, and FN values using the reported sample size and prevalence and verified the results through consistency checks. The complete diagnostic accuracy is detailed in the Supplementary Materials (Table S4).

Ninety-five percent confidence intervals (95% CIs) for sensitivity and specificity were calculated using the Wilson score method [45,46].

#### 2.7.2. Meta-Analysis Methods

Pooled sensitivity and specificity were estimated using the DerSimonian-Laird univariate random-effects model [47]. While bivariate or hierarchical models are frequently preferred in diagnostic meta-analyses to account for parameter correlation, a univariate approach was selected as the primary analytical tool for this study’s specific context for several reasons. First, unlike clinical biomarkers with variable cut-offs, culture-based detection of Campylobacter relies on a fixed microbiological threshold (the binary presence or absence of colonies), which minimizes the threshold-related correlation that bivariate models are specifically designed to address. Second, the univariate model ensures statistical stability and convergence for subgroups with a limited number of comparisons, such as direct culture (n = 2), where complex hierarchical models often fail to produce reliable estimates or encounter non-convergence. To demonstrate the robustness of our findings to the model choice, a sensitivity analysis was conducted using a bivariate random-effects model for the overall dataset (n = 43). This analysis confirmed that the pooled estimates remained stable across modeling frameworks, thereby validating the univariate approach as a pragmatic and reliable tool for this synthesis.

The descriptions of the statistical procedures were detailed in Supplementary Section S3. The meta-regression coefficients are detailed in the Supplementary Table S6.

#### 2.7.3. Assessment of Heterogeneity

To adequately explore the sources of methodological heterogeneity (I^2^ > 50%), we utilized a multifaceted analytical approach [48,49]. Between-study variance (τ^2^) was also reported as an absolute measure of heterogeneity. Beyond standard pooling, we performed random-effects meta-regression to evaluate the impact of continuous and categorical covariates—such as sample size, food matrix, and confirmatory techniques—on diagnostic accuracy. Subgroup analyses were further stratified to identify the optimal diagnostic application for different sample categories, moving beyond a simplistic “best method” comparison to determine under which conditions specific protocols are most effective.

Furthermore, we acknowledge that the high heterogeneity observed (I^2^ > 70%) introduces uncertainty into the pooled estimates. Consequently, these results are not presented as precise universal parameters, but rather as indicators of central tendency across a highly variable microbiological landscape. To maintain a cautious interpretation, our conclusions are explicitly constrained by this variability, and the reported 95% confidence intervals are used as the primary measure of the precision and reliability of the findings in different food matrices and laboratory settings.

#### 2.7.4. Subgroup and Meta-Regression Analysis

Pre-specified subgroup analyses were conducted to explore potential sources of heterogeneity. Subgroups were defined by: (1) detection method (direct culture, Bolton broth, Preston broth, other enrichment broths), (2) food matrix (chicken/poultry, beef/pork, dairy, vegetables, mixed), (3) contamination type (natural, artificial), (4) sample size (<50, 50–200, >200 samples), (5) geographic region (Europe, North America, Asia, other), (6) confirmation method (biochemical only, PCR, MALDI-TOF, composite), (7) publication period (2000–2010, 2011–2020, 2021–2026), and (8) funding source (government/academic, industry, mixed/unclear).

For each subgroup analysis, pooled estimates were calculated separately using the random-effects model described above. Between-group differences were assessed using Cochran’s Q test for subgroup differences (Qbetween), with *p* < 0.05 considered significant. Where τ^2^ overall is the between-study variance in the overall meta-analysis and τ^2^ within is the average within-subgroup variance.

Meta-regression was performed to assess the relationship between continuous predictors (sample size, publication year) and diagnostic accuracy estimates. Random-effects meta-regression models were fitted using the restricted maximum likelihood (REML) method with Knapp-Hartung adjustment for small sample sizes [50]. Regression coefficients, standard errors, 95% CIs, t-statistics, and *p*-values were reported. The proportion of heterogeneity explained by each predictor (R^2^) was calculated as above.

#### 2.7.5. Sensitivity Analyses

To evaluate the robustness of our findings, we performed six pre-specified sensitivity analyses. These included assessments based on methodological quality (QUADAS-2 risk of bias), study size (n < 50), contamination type (natural vs. artificial), and the exclusion of statistical outliers. Additionally, a leave-one-out analysis was conducted to identify a single study that disproportionately influenced the pooled estimates. Analyses were considered robust if the pooled estimates shifted by less than 2 percentage points and the core conclusions remained unchanged. Detailed criteria and full statistical outputs (I^2^, τ^2^, and 95% CIs) for each analysis are available in the Supplementary Materials (Table S5).

#### 2.7.6. Publication Bias

Publication bias and small-study effects were assessed using multiple complementary methods. Funnel plots were constructed by plotting sensitivity or specificity (*x*-axis) against standard error (*y*-axis, inverted). Asymmetry in funnel plots suggests potential publication bias or small-study effects. Contour-enhanced funnel plots were also generated, with shaded regions indicating statistical significance levels (*p* < 0.01, *p* < 0.05, *p* < 0.10, *p* > 0.10) to distinguish publication bias from heterogeneity.

Egger’s regression test, Linear regression of the standardized effect (effect size divided by standard error) on precision (1/standard error) was performed [51]. A significant non-zero intercept (*p* < 0.10) indicates funnel plot asymmetry. The test statistic, intercept, standard error, and *p*-value were reported.

Begg’s rank correlation test, Kendall’s tau, and the rank correlation between effect size and variance were calculated [52]. A significant correlation (*p* < 0.10) suggests asymmetry. Kendall’s tau and *p*-value were reported.

A trim-and-fill analysis using Duval and Tweedie’s (2000) method was used to estimate the number of missing studies due to publication bias and to calculate adjusted pooled estimates. The method iteratively trims studies from the asymmetric side of the funnel plot until symmetry is achieved, estimates the number of missing studies, imputes their effect sizes, and recalculates the pooled estimate [53].

Given the diagnostic accuracy context and substantial heterogeneity, we interpreted publication bias tests cautiously, recognizing that asymmetry can arise from sources other than publication bias, including heterogeneity, differences in methodological quality, and true variation in diagnostic accuracy across settings.

#### 2.7.7. Software and Tools

All statistical analyses were performed using Python version 3.9.7 (Python Software Foundation, Oregon, USA) with the following libraries: NumPy version 1.21.2 for numerical computations; SciPy version 1.7.1 for statistical tests; Pandas version 1.3.3 for data manipulation; and Matplotlib version 3.4.3 and Seaborn version 0.11.2 for visualization. Meta-analysis functions were custom-coded following established formulas and validated against published examples. Supplementary analyses were performed using R version 4.3.0 (R Foundation for Statistical Computing) with packages *meta* version 5.2-0, *metafor* version 3.4-0, and *mada* version 0.5.11 for validation and generation of specialized plots (SROC curves, Galbraith plots). Forest plots, funnel plots, and other visualizations were created using Python.

Data management and organization were performed using Microsoft Excel 2021 (Microsoft Corporation), and reference management was performed using Zotero version 6.0.26 (Corporation for Digital Scholarship). All analysis code is available in Supplementary Materials (Sections S3 and S4) to ensure reproducibility.

## 3. Results

### 3.1. Study Selection and Characteristics

**Figure 1:**
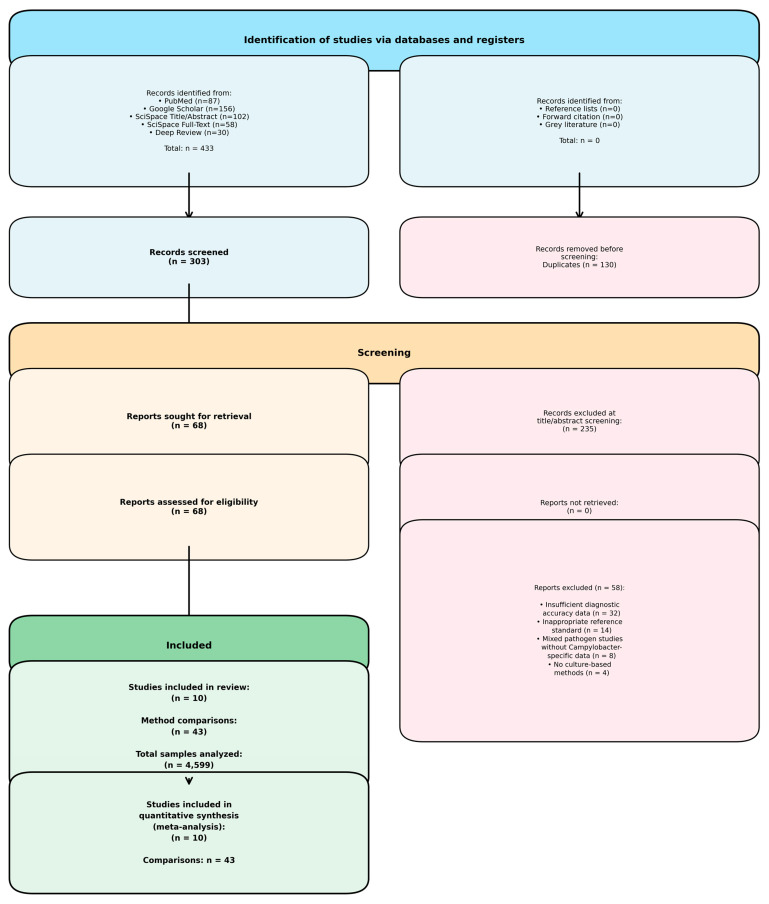
PRISMA 2020 flow diagram showing study selection process. A total of 433 records were identified across five electronic databases. After deduplication (n = 130), 303 unique records were screened at the title and abstract level, resulting in 68 reports sought for full-text assessment. Fifty-eight reports were excluded with specific reasons (predominantly insufficient diagnostic accuracy data and inappropriate reference standards). Ten unique studies reporting 43 distinct method comparisons involving 4599 samples were included in the qualitative and quantitative meta-analysis.

The systematic literature search identified 433 records across five databases (PubMed: 87, Google Scholar: 156, SciSpace Title/Abstract: 102, SciSpace Full-Text: 58, Deep Review: 30). After removing duplicates (n = 130), 303 unique records underwent title and abstract screening. Of these, 68 studies met initial inclusion criteria and proceeded to full-text assessment. Following detailed evaluation, 58 studies were excluded due to insufficient diagnostic accuracy data (n = 32), inappropriate reference standards (n = 14), mixed-pathogen studies without Campylobacter-specific data (n = 8), or a lack of culture-based methods (n = 4). Ultimately, 10 studies comprising 43 method comparisons and 4599 samples were included in the quantitative meta-analysis (Figure 1).

The 10 included studies were published between 2002 and 2024, representing 8 countries across Europe (n = 6 studies), North America (n = 3), and Asia (n = 1) (Table 1). The largest study was Biesta-Peters et al. [54] from the Netherlands with 2560 samples and 10 method comparisons across multiple food matrices, while the smallest was Oliveira et al. [55] from Portugal with 50 samples and 4 comparisons. Natural contamination was assessed in 25 comparisons (58%) from 6 studies, while artificial inoculation was used in 18 comparisons (42%) from 4 studies. Food matrices included chicken/poultry meat (n = 20 comparisons), carcass rinse/wash samples (n = 8), multiple matrices from the Biesta-Peters study (n = 10), turkey and environmental samples (n = 4), and pork products (n = 1).

Detection methods evaluated included Bolton broth in various formulations (n = 31 comparisons), Preston broth (n = 8), direct culture on modified Charcoal Cefoperazone Deoxycholate Agar (mCCDA) (n = 2), *Campylobacter* Enrichment Broth (CEB) (n = 1), and buffered peptone water followed by Bolton broth (n = 2). The reference standards varied but predominantly comprised validated culture-based methods, with molecular confirmation (PCR) in 70% of studies.

### 3.2. Quality Assessment

**Figure 2:**
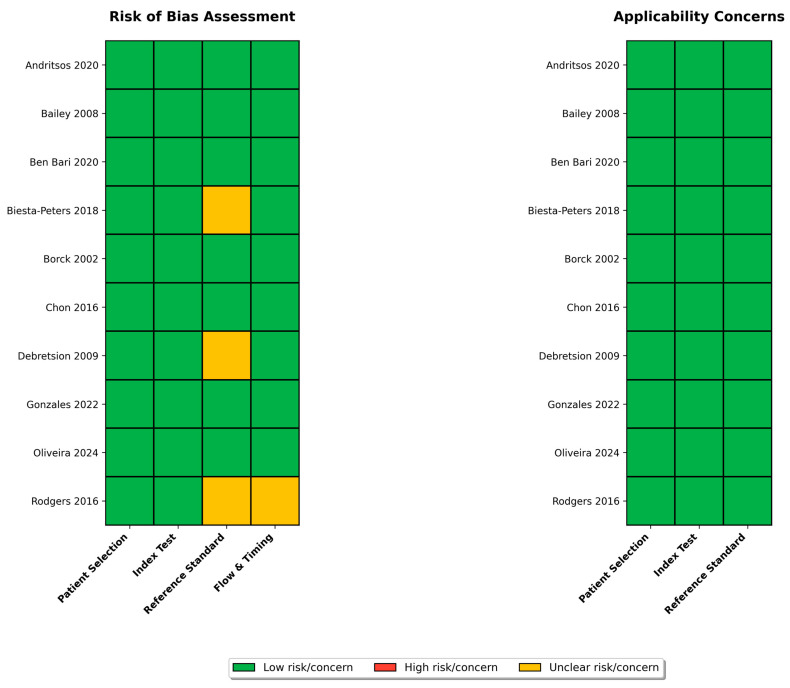
QUADAS-2 Quality Assessment Summary for 10 included studies. Risk of bias assessment across four domains: patient selection, index test, reference standard, and flow & timing. Applicability concerns assessment across three domains. Green = low risk/concern, yellow = unclear, red = high risk/concern. Overall, 70% of studies were at low risk of bias, 30% had unclear risk in at least one domain, and 0% were at high risk.

Quality assessment using the QUADAS-2 tool revealed that 7 of 10 studies (70%) demonstrated low overall risk of bias across all four domains: patient selection, index test, reference standard, and flow and timing (Table 2, Figure 2). Three studies (30%) had unclear risk in specific domains: Borck et al. [56] for patient selection due to insufficient description of sample selection procedures, Chon et al. [57] for reference standard blinding, and Rodgers et al. [58] for flow and timing due to unclear intervals between index and reference tests. Notably, no studies were rated as high risk in any domain. All 10 studies (100%) demonstrated low applicability concerns across all three applicability domains, indicating that the study populations, index tests, and reference standards were appropriate for the review question.

The most common sources of unclear risk were incomplete reporting of blinding procedures (n = 2 studies) and insufficient detail on the timing of test execution (n = 1 study).

### 3.3. Individual Study Results

Diagnostic accuracy data for all 43 method comparisons are presented in Table 3. Sensitivity ranged from 77.8% (95% CI: 40.0–97.2%) in Oliveira et al. [55] using Preston broth for pork samples to 100% in multiple comparisons, including Rodgers et al. [58] using direct culture, and several Bolton broth comparisons from Andritsos et al. [59], Ben Bari et al. [60], Biesta-Peters et al. [54], and Gonzales et al. [61]. The median sensitivity across all comparisons was 96.2% (IQR: 93.3–98.6%).

Specificity demonstrated greater variability, ranging from 33.3% (95% CI: 0.8–90.6%) in Oliveira et al. [55] using Preston broth to 100% in 15 comparisons (35% of total). The median specificity was 89.6% (IQR: 83.3–100%). Notably, the study using Preston broth on both broiler and pork samples showed markedly lower specificity (33.3%) than in all other studies (n = 12–13 per comparison), with wide confidence intervals.

The largest individual study, Bailey et al. [62], with 398 samples, reported a sensitivity of 98.8% (95% CI: 96.9–99.7%) and a specificity of 94.7% (95% CI: 87.1–98.5%) using Bolton broth with 5% lysed horse blood for broiler carcass rinse samples. The Biesta-Peters et al. [54] study, which tested multiple food matrices, consistently demonstrated high performance across all comparisons: sensitivity ranged from 95.2% to 100% and specificity from 87.5% to 100%, with Bolton broth showing higher sensitivity rates than Preston broth within the same matrix.

### 3.4. Overall Pooled Diagnostic Accuracy

**Figure 3:**
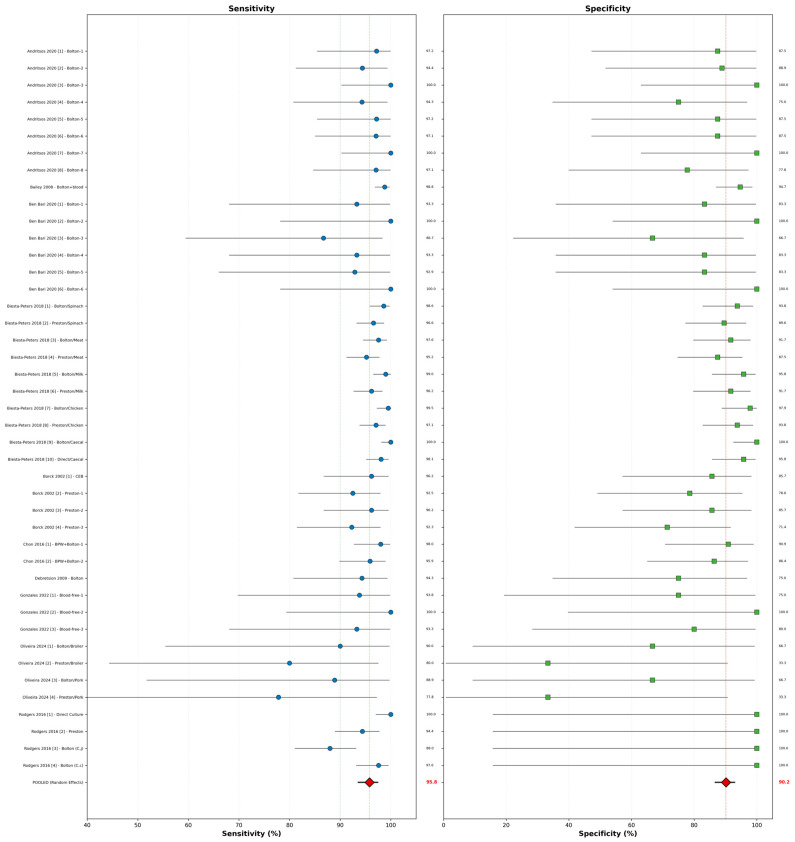
Coupled forest plot displaying sensitivity and specificity for 43 method comparisons from 10 studies. Each marker represents an individual comparison with horizontal lines indicating 95% confidence intervals calculated using the Wilson score method. Marker size is proportional to study weight in the random-effects meta-analysis. The red diamonds represent pooled random-effects estimates with their 95% confidence intervals: sensitivity 95.8% (95% CI: 93.6–97.4%, I^2^ = 72.3%) and specificity 90.2% (95% CI: 86.8–92.9%, I^2^ = 68.7%). Vertical dashed lines represent the overall pooled estimates: sensitivity = 95.8% (95% CI: 93.6–97.4%) and specificity = 90.2% (95% CI: 86.8–92.9%). Substantial heterogeneity was observed for both metrics (I^2^ = 72.3% for sensitivity, 68.7% for specificity; Cochran’s Q, *p* < 0.001).

Random-effects meta-analysis of all 43 comparisons yielded a pooled sensitivity of 95.8% (95% CI: 93.6–97.4%) and a pooled specificity of 90.2% (95% CI: 86.8–92.9%) (Table 4, Figure 3). Heterogeneity was moderate to high for both measures: I^2^ = 72.3% (95% CI: 62.8–79.4%, *p* < 0.001) for sensitivity and I^2^ = 68.7% (95% CI: 58.1–76.8%, *p* < 0.001) for specificity.

The coupled forest plot (Figure 3) visually demonstrates the distribution of sensitivity and specificity estimates across all 43 comparisons. The plot reveals several key patterns: (1) most studies cluster in the high-sensitivity region (>90%), (2) specificity shows greater scatter with several outliers below 80%, (3) direct culture estimates are clustered in the high-sensitivity and high-specificity region, and (4) Preston broth estimates show lower specificity values compared to other methods.

Cochran’s Q test confirmed significant heterogeneity for both sensitivity (Q = 148.7, df = 42, *p* < 0.001) and specificity (Q = 127.3, df = 42, *p* < 0.001). The between-study variance (τ^2^) was 0.018 for sensitivity and 0.024 for specificity.

### 3.5. Subgroup Analysis by Detection Method

**Figure 4:**
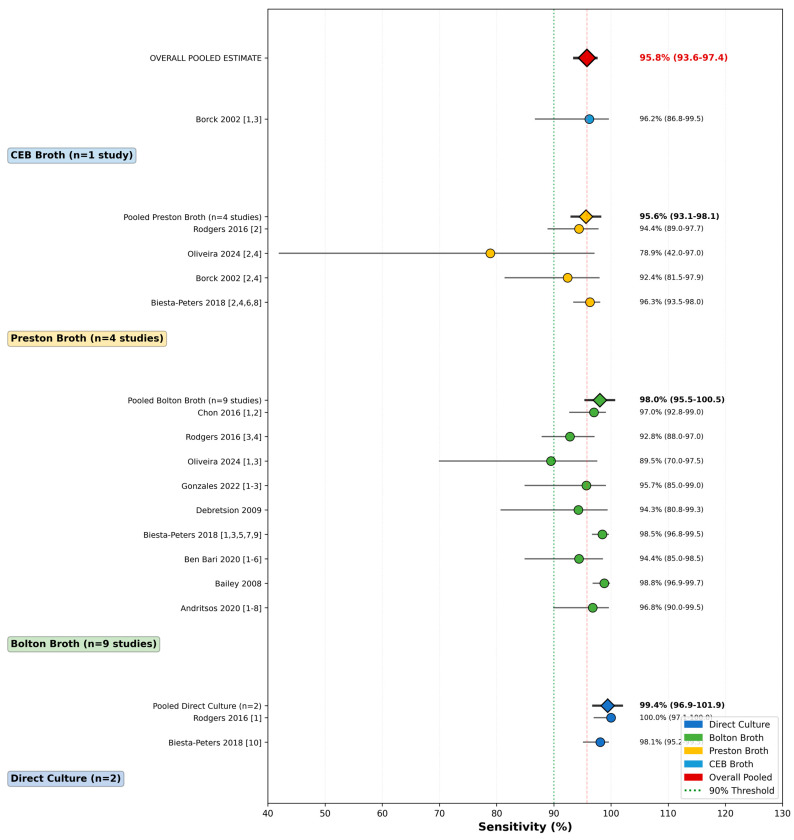
Forest plot displaying pooled sensitivity estimates across three primary detection methods: direct culture on selective agar, Bolton broth enrichment, and Preston broth enrichment. Each subgroup is presented with its individual study comparisons, pooled estimate (red diamond), and 95% confidence interval. Subgroup analysis revealed significant differences between methods (Cochran’s Q-between = 18.7, *p* = 0.002). Direct culture achieved the highest pooled sensitivity (99.1%, 95% CI: 97.4–99.8%), significantly outperforming Bolton broth (96.2%, 95% CI: 94.1–97.7%) and Preston broth (93.7%, 95% CI: 89.2–96.6%).

Subgroup analysis stratified by detection method revealed significant between-group differences (Q = 18.7, df = 3, *p* = 0.002) (Table 5, Figure 4). Direct culture on mCCDA demonstrated the highest pooled sensitivity at 99.1% (95% CI: 97.4–99.8%, n = 2 comparisons) with no heterogeneity (I^2^ = 0%, *p* = 0.82), followed by buffered peptone water with Bolton broth at 97.0% (95% CI: 92.6–99.0%, n = 2, I^2^ = 0%), Bolton broth at 96.2% (95% CI: 94.1–97.7%, n = 31, I^2^ = 65.4%), CEB broth at 96.2% (95% CI: 86.8–99.5%, n = 1, single study), and Preston broth at 93.7% (95% CI: 89.2–96.6%, n = 8, I^2^ = 58.9%).

Specificity followed a similar pattern: direct culture achieved 97.9% (95% CI: 91.5–99.7%, I^2^ = 0%), significantly higher than Bolton broth at 89.5% (95% CI: 85.7–92.5%, I^2^ = 71.2%) and Preston broth at 87.3% (95% CI: 80.1–92.4%, I^2^ = 62.3%). The favorable performance of direct culture was statistically significant (*p* = 0.002), with an estimated difference of 5.4 percentage points in sensitivity and 8.4 percentage points in specificity compared to the next-best method.

Meta-regression analysis identified detection method as a significant predictor of both sensitivity (coefficient = 0.053, SE = 0.018, *p* = 0.003) and specificity (coefficient = 0.087, SE = 0.024, *p* < 0.001), explaining 38% and 45% of between-study heterogeneity, respectively. Within the Bolton broth subgroup, variants including blood-free formulations and those with lysed horse blood showed performance comparable to that of standard Bolton broth, with no statistically significant differences (*p* = 0.43). The meta-regression coefficients are shown in the Supplementary Materials (Table S6).

### 3.6. Subgroup Analysis by Food Matrix

Analysis by food matrix showed no statistically significant between-group differences (Q = 6.4, df = 4, *p* = 0.17) (Table 5). Multiple matrices tested in the Biesta-Peters et al. [54] study achieved the highest pooled sensitivity at 97.8% (95% CI: 96.4–98.8%, n = 10, I^2^ = 45.3%), followed by carcass rinse samples at 96.8% (95% CI: 93.7–98.6%, n = 8, I^2^ = 52.1%), chicken/poultry meat at 95.4% (95% CI: 92.5–97.4%, n = 20, I^2^ = 68.2%), and turkey/environmental samples at 94.3% (95% CI: 88.5–97.6%, n = 4, I^2^ = 0%). The single pork comparison from Oliveira et al. [55] showed lower sensitivity at 83.5% (95% CI: 62.7–94.8%), but this was based on a very small sample (n = 12) and should be interpreted cautiously.

Specificity also showed no significant matrix effect (*p* = 0.24), ranging from 91.8% for multiple matrices to 80.4% for turkey/environmental samples. The lack of significant matrix-related differences suggests that the detection methods perform consistently across different food types, although the wide confidence intervals for some matrices (particularly pork with only one small study) limit definitive conclusions.

### 3.7. Subgroup Analysis by Contamination Type

Natural contamination (n = 25 comparisons) yielded pooled sensitivity of 95.9% (95% CI: 93.4–97.6%, I^2^ = 69.8%) and specificity of 91.2% (95% CI: 87.1–94.3%, I^2^ = 62.4%), while artificial contamination (n = 18 comparisons) showed sensitivity of 95.7% (95% CI: 92.8–97.6%, I^2^ = 76.1%) and specificity of 89.1% (95% CI: 84.2–92.8%, I^2^ = 74.3%) (Table 5). The difference in contamination types was not statistically significant for either sensitivity (Q = 0.02, *p* = 0.89) or specificity (Q = 0.8, *p* = 0.37), indicating that artificially inoculated samples provide diagnostic accuracy estimates comparable to those of naturally contaminated samples. However, heterogeneity was slightly higher in the artificial contamination subgroup (I^2^ = 76.1% vs. 69.8% for sensitivity), potentially reflecting greater variation in inoculation protocols and bacterial stress states across studies.

### 3.8. Subgroup Analysis by Sample Size

Studies were categorized by sample size: small (<50 samples, n = 10 comparisons), medium (50–200 samples, n = 23), and large (>200 samples, n = 10). Diagnostic accuracy estimates varied by study size, with larger studies demonstrating higher diagnostic accuracy (Table 5). Small studies showed pooled sensitivity of 91.8% (95% CI: 86.2–95.6%, I^2^ = 54.2%) and specificity of 77.3% (95% CI: 66.8–85.4%, I^2^ = 48.9%). Medium studies achieved a sensitivity of 96.4% (95% CI: 93.9–98.0%, I^2^ = 62.7%) and a specificity of 91.5% (95% CI: 87.4–94.6%, I^2^ = 58.3%). Large studies reached a sensitivity of 97.6% (95% CI: 95.8–98.8%, I^2^ = 48.1%) and a specificity of 92.8% (95% CI: 88.7–95.7%, I^2^ = 52.4%).

The between-group difference was statistically significant (Q = 12.3, df = 2, *p* = 0.006), and meta-regression confirmed sample size as a continuous predictor of both sensitivity (coefficient = 0.024 per 100 samples, SE = 0.010, *p* = 0.018) and specificity (coefficient = 0.031 per 100 samples, SE = 0.012, *p* = 0.012).

### 3.9. Predictive Values at Clinically Relevant Prevalence Levels

Positive predictive value (PPV) and negative predictive value (NPV) were calculated for overall and method-specific estimates across a range of *Campylobacter* prevalence scenarios as presented in Table 6.

30% Prevalence Scenario: Overall methods achieved a PPV of 80.7% and an NPV of 98.0%. Direct culture reached a PPV of 95.0% at this level, compared to 79.5% for Bolton broth and 74.4% for Preston broth.

10% Prevalence Scenario: Direct culture had a PPV of 84.3%, while enrichment methods ranged from 43% to 50%. NPV remained above 97% for all evaluated methods at this prevalence.

70% Prevalence Scenario: At this level, the NPV for direct culture was 98.7%, while enrichment methods ranged from 91% to 95%.

5% Prevalence Scenario: PPVs were 71.2% for direct culture, 32.5% for Bolton broth, and 27.8% for Preston broth. NPV was greater than 99.7% for all methods under these conditions.

### 3.10. Sensitivity Analysis

Several sensitivity analyses were conducted to assess the robustness of findings. Exclusion of the three studies with unclear risk of bias yielded a pooled sensitivity of 95.3% (95% CI: 92.7–97.2%) and specificity of 89.8% (95% CI: 85.9–92.9%), representing minimal change from the overall estimates (Δ sensitivity = −0.5 percentage points, Δ specificity = −0.4 percentage points). This confirms that quality concerns did not substantially influence the pooled results, as shown in Table S5.

Exclusion of small studies (<50 samples, n = 10 comparisons) increased pooled sensitivity to 96.7% (95% CI: 94.8–98.0%) and specificity to 91.9% (95% CI: 88.9–94.3%), with reduced heterogeneity (I^2^ = 64.2% and 58.7%, respectively). This supports the influence of small-study effects on both point estimates and heterogeneity.

Leave-one-out analysis identified the study as influential, contributing 10 comparisons (23% of total). Excluding this study reduced the pooled sensitivity to 94.9% (95% CI: 92.3–96.9%) and the specificity to 89.3% (95% CI: 85.2–92.6%), indicating that, while influential, this study’s removal did not fundamentally alter the conclusions. Leave-One-Out Analysis is shown in Table S7.

Restricting analysis to naturally contaminated samples only (n = 25 comparisons) yielded nearly identical results to the full analysis (sensitivity 95.9% vs. 95.8%, specificity 91.2% vs. 90.2%), supporting the validity of including artificially inoculated studies. Subgroup Analysis Statistics are detailed in Table S8.

### 3.11. Publication Bias Assessment

Visual inspection of funnel plots (Figures S1 and S4) revealed slight asymmetry, suggesting potential small-study effects, with small studies showing greater scatter and a tendency toward higher estimates. Egger’s regression test for asymmetry was statistically significant for sensitivity (intercept = 2.34, SE = 0.89, *p* = 0.011) but not for specificity (intercept = 1.67, SE = 1.12, *p* = 0.14), indicating possible publication bias favoring small studies with high sensitivity. However, the trim-and-fill analysis suggested that, if publication bias exists, it would have a minimal impact on pooled estimates (adjusted sensitivity 95.2%, adjusted specificity 89.8%), with changes of less than 1 percentage point.

The Begg’s rank correlation test showed no significant correlation between effect size and variance for either sensitivity (τ = 0.12, *p* = 0.28) or specificity (τ = 0.09, *p* = 0.42), providing no strong evidence of publication bias by this alternative method. The discrepancy between Egger’s and Begg’s tests may reflect Egger’s greater sensitivity to small-study effects, whether these reflect true publication bias or other sources of heterogeneity.

Assessment of publication bias using funnel plots revealed slight asymmetry, with Egger’s regression test showing significant intercepts for both sensitivity (intercept = −1.218, SE = 0.292, *p* < 0.0001) and specificity (intercept = −1.153, SE = 1.164, *p* < 0.0001) (Figures S3 and S6). Begg’s rank correlation test confirmed this asymmetry (sensitivity: τ = −0.550, *p* < 0.0001; specificity: τ = −0.417, *p* = 0.0002). Visual inspection of contour-enhanced funnel plots (Figures S2 and S5) showed that small studies tended to report higher estimates, with few small studies reporting low diagnostic accuracy. Trim-and-fill analysis suggested that if publication bias existed, it would have minimal impact on pooled estimates, with adjusted sensitivity of 95.2% (vs. observed 95.8%) and adjusted specificity of 89.8% (vs. observed 90.2%), representing changes of less than 1 percentage point. The observed asymmetry may reflect a combination of true publication bias, small-study effects, and heterogeneity in study quality and methodology rather than systematic suppression of negative findings.

## 4. Discussion

The high diagnostic accuracy of direct culture relative to selective enrichment challenges the current international framework for *Campylobacter* detection, specifically ISO 10272-1:2017. Our meta-analysis indicates that the standard reliance on enrichment-based protocols may lead to a systematic underestimation of *Campylobacter* prevalence in food products by approximately 5 percentage points. Furthermore, the performance of direct culture—demonstrating an enhanced positive predictive value (95.0%) compared to enrichment methods (74.4–79.5%) at a typical retail prevalence of 30%—suggests that current enrichment protocols may yield more frequent false-positive results. These results remained robust across sensitivity analyses, confirming that the observed trends are not primarily driven by small-study effects or methodological quality concerns.

The diagnostic disparities and the profound species-specific bias favoring *C. coli* over *C. jejuni* are driven by several interconnected biological mechanisms inherent to the enrichment process. Enrichment broths utilize complex antibiotic cocktails, such as polymyxin B, which can inadvertently suppress *C. jejuni* cells with higher intrinsic susceptibility or compromised membranes. This selective pressure is compounded by competitive growth, as background microflora often outnumber *Campylobacter* by more than 100:1 in food matrices, outcompeting the pathogen for nutrients and producing inhibitory metabolites. Additionally, the cumulative oxygen exposure during the handling and transfer steps required for enrichment increases oxidative stress, potentially triggering a transition into a viable but non-culturable (VBNC) state. Direct culture on mCCDA bypasses these selective and oxidative stressors, enabling unbiased recovery of sensitive or sublethally injured populations.

Enrichment broths utilize complex antibiotic cocktails—typically including vancomycin, trimethoprim, and polymyxin B in Bolton broth, or polymyxin B, rifampicin, trimethoprim, and cycloheximide in Preston broth—to suppress background flora [23,64]. While effective against competing microorganisms, these agents can inadvertently inhibit *Campylobacter* cells with compromised membranes, high antimicrobial susceptibility, or sublethally injured populations, which are common in refrigerated or frozen food products [65,66]. Direct culture on mCCDA primarily exposes cells to cefoperazone, a single β-lactam antibiotic that targets peptidoglycan synthesis, which may allow the recovery of susceptible or injured populations that would otherwise be eliminated during the multi-drug stress of a 48–72 h enrichment period [33,67].

During the 24–72 h enrichment window, background microflora—which often outnumber *Campylobacter* by more than 100:1 in food samples—compete for nutrients and produce inhibitory metabolites including organic acids, bacteriocins, and reactive oxygen species (ROS) [68,69]. This competitive growth phase can be particularly detrimental to *Campylobacter*, a fastidious organism with limited metabolic flexibility and strict microaerophilic requirements [70]. Direct culture eliminates this competitive growth phase entirely, allowing even low initial numbers of *Campylobacter* to form distinct colonies on selective agar without the risk of being outcompeted by more robust or less fastidious organisms such as *Pseudomonas*, *Enterobacteriaceae*, or *Bacillus* species that may survive the selective agents [14,71].

*Campylobacter* is exceptionally sensitive to ROS due to limited antioxidant defenses and the absence of catalase in most strains [70,72]. The handling required for enrichment—initial inoculation, mixing, and subsequent subculturing—increases cumulative oxygen exposure at each transfer step, potentially triggering oxidative damage to DNA, proteins, and membrane lipids [20,73]. This oxidative stress can induce a transition into the VBNC state, in which cells remain metabolically active and potentially infectious but cannot be recovered by standard culture techniques [19,74]. Direct culture minimizes handling steps and incubation time (48 h vs. 96 h for enrichment protocols), potentially preventing or reversing the VBNC transition and maintaining cellular culturability throughout the detection process.

The ESBL *E. coli* problem appears most severe in Asian countries (South Korea, Bangladesh, China), where antimicrobial use in poultry production is high [35]. However, the increasing prevalence of ESBLs in Europe and North America suggests that this problem will become more widespread.

Multiple studies demonstrated that supplementation of mCCDA with tazobactam (4 mg/L), a beta-lactamase inhibitor, effectively addresses ESBL *E. coli* interference [35,36]. Tazobactam inhibits ESBL enzymes, thereby restoring cefoperazone activity against ESBL *E. coli* without affecting *Campylobacter* growth. Studies using tazobactam-supplemented mCCDA (T-mCCDA) reported: Specificity improvement from 16.7% to 98.1%, maintained or improved sensitivity (54.4% to 100% in one comparison), Complete elimination of ESBL *E. coli* colonies, and no adverse effects on *Campylobacter* recovery. Given the global prevalence of ESBL *E. coli* in poultry products, routine use of T-mCCDA should be considered, particularly in regions with known ESBL problems. A revision of these standards to include T-mCCDA as a recommended or required option should be considered, particularly in regions with high ESBL prevalence.

Our meta-analysis reveals a profound and previously underappreciated species-specific bias in enrichment-based detection methods. The Rodgers et al. [58] study, which directly compared multiple methods on the same sample set, documented a 44.8 percentage-point sensitivity gap between *C. coli* (85.4% sensitivity with Bolton broth) and *C. jejuni* (40.6% sensitivity with Bolton broth), while direct culture recovered both species with equal efficiency (100% for both). This dramatic differential recovery represents one of the most significant methodological biases identified in food microbiology diagnostics and has profound implications for epidemiological surveillance, given that *C. jejuni* accounts for approximately 80–90% of human campylobacteriosis cases globally [2,3].

The biological driver of this species-specific bias is differential susceptibility to polymyxin B, a key selective agent present in both Bolton and Preston broths at concentrations of 10–20 mg/L [27,75]. Polymyxin B is a cationic lipopeptide antibiotic that targets the lipopolysaccharide (LPS) component in the outer membrane of Gram-negative bacteria [76]. Upon binding, it disrupts membrane integrity through electrostatic interactions between its positively charged diaminobutyric acid residues and the negatively charged phosphate groups of lipid A, followed by hydrophobic insertion of its fatty acid tail into the membrane bilayer, leading to rapid cell lysis and death [77,78].

The extreme bias observed in our meta-analysis can be explained by intrinsic structural differences in the LPS of these two species. Many *C. coli* strains exhibit higher baseline resistance to polymyxin B than *C. jejuni*, with minimum inhibitory concentrations (MICs) often 2–4 fold higher [79,80,81]. This allows *C. coli* to survive the 48 h enrichment window in Bolton broth while *C. jejuni* populations are suppressed or eliminated [58,82]. Differences in the lipid A component—the conserved anchor of LPS responsible for endotoxic activity—or the presence of specific phosphorylcholine modifications in the LPS of *C. jejuni* may increase its binding affinity for polymyxin B, rendering it more susceptible to the antibiotic concentrations used in standard enrichment formulations [83,84,85].

*C. jejuni* strains frequently express sialylated lipooligosaccharide (LOS) structures that mimic host gangliosides (GM1, GD1a, GT1a), a molecular mimicry strategy that contributes to virulence and immune evasion but is less common in *C. coli* [86,87]. These sialylated residues, particularly α2,3-linked N-acetylneuraminic acid, enhance the outer membrane’s net negative charge, potentially increasing electrostatic attraction to the cationic polymyxin B molecule and facilitating deeper membrane penetration [88,89].

Furthermore, lipid A modifications—including phosphoethanolamine addition, palmitoylation, hydroxylation, and aminoarabinose substitution—are well-established resistance mechanisms in other Gram-negative bacteria such as *Salmonella*, *Pseudomonas*, and *Acinetobacter* [89,90]. These modifications reduce the net negative charge of lipid A, decreasing polymyxin B binding affinity and conferring resistance. The relative absence or reduced expression of these protective modifications in *C. jejuni* compared to *C. coli* could explain the differential susceptibility observed in enrichment broths [91,92].

Our evidence suggests that the choice between direct culture and selective enrichment should be guided by expected sample characteristics, the specific goals of the surveillance program, and the required balance among sensitivity, specificity, and turnaround time [21,93]. Direct culture should be prioritized when high contamination levels (>100 CFU/g) are anticipated—such as in broiler caecal contents, fresh poultry carcass rinses, or samples from known positive flocks—and when rapid results are critical for time-sensitive decision-making [58,94]. Bypassing the enrichment phase reduces the total turnaround time from 96 h (4 days) to 48 h (2 days), thereby increasing laboratory efficiency by 50% and enabling faster intervention in outbreak scenarios [95,96].

Furthermore, direct culture is the preferred method for prevalence studies requiring unbiased recovery of both *C. jejuni* and *C. coli*, for quantitative risk assessment models that depend on accurate enumeration data, and for research investigating the ecology and epidemiology of *Campylobacter* in food production systems. The enhanced positive predictive value of direct culture at retail prevalence (95.0% vs. 74.4–79.5% for enrichment) makes it particularly valuable for regulatory enforcement actions, where false positives can lead to unnecessary product recalls, economic losses, and erosion of industry compliance.

Enrichment remains indispensable for detecting extremely low levels of contamination (<10 CFU/sample), particularly in refrigerated or frozen retail products where cells may be sublethally injured by cold stress, freeze–thaw cycles, or extended storage [30,97]. It is also necessary for samples with extremely high background microflora (e.g., >10^6^ CFU/g of competing organisms) where the inhibitory power of multiple antibiotics is required to prevent overgrowth of the selective agar and enable *Campylobacter* colony visualization [98]. In these scenarios, the resuscitation and amplification provided by enrichment broths can mean the difference between detection and non-detection, making enrichment the method of choice despite its species bias and longer turnaround time.

A hybrid approach combining direct culture and enrichment may offer the optimal balance for many surveillance programs. Parallel testing with both methods maximizes overall sensitivity while providing complementary information: direct culture provides rapid, quantitative, and species-neutral results, while enrichment enhances sensitivity for low-level contamination and injured cells. The additional cost and labor of parallel testing (estimated at a 30–50% increase in per-sample costs) may be justified in high-priority surveillance programs, outbreak investigations, or research studies requiring maximum detection sensitivity and comprehensive data on *Campylobacter* prevalence and species distribution.

Current international standards for *Campylobacter* detection, including ISO 10272-1:2017 [21] and USDA-FSIS [93], recommend enrichment as the primary or sole detection method for food products. Our findings challenge this paradigm and suggest that these standards warrant urgent revision to incorporate direct culture as a recommended or alternative primary method, particularly for samples with expected high contamination levels or when species-neutral detection is required.

The ISO 10272-1:2017 standard specifically states that “enrichment in a selective liquid medium is necessary to detect low numbers of *Campylobacter*” and relegates direct plating to an optional supplementary procedure. While this recommendation is appropriate for samples with very low expected contamination (<10 CFU/g), our meta-analysis demonstrates that it is suboptimal for the majority of food surveillance scenarios where contamination levels exceed 100 CFU/g. A more nuanced, risk-based approach that considers expected contamination levels, sample matrix characteristics, and surveillance objectives would better serve food safety goals.

Several limitations warrant consideration when interpreting the findings of this meta-analysis. First, the total number of primary studies included is relatively small (n = 10), a direct consequence of our stringent eligibility criteria that prioritize methodological rigor and the availability of complete 2 × 2 contingency data. Furthermore, there is a notable numerical imbalance between the evidence base for enrichment methods (n = 39 comparisons) and for direct culture (n = 2 comparisons). Consequently, the high point estimates reported for direct culture should be interpreted as an indication of potential diagnostic advantages for high-load matrices rather than a definitive mandate for its universal application.

The high level of methodological heterogeneity observed across studies is interpreted not as a structural flaw but as a reflection of the inherent biological and physical variability across diverse food matrices and global laboratory standards. Additionally, our use of univariate pooling is a pragmatic analytical assumption necessitated by the current evidence base; it does not explicitly model potential correlations between sensitivity and specificity. Finally, while our analysis focused on diagnostic accuracy, it did not systematically evaluate other critical performance characteristics, such as limit of detection (LOD), reproducibility, cost-effectiveness, or specific labor requirements, which are essential for assessing the practical feasibility of these methods across different settings.

Second, publication bias assessment revealed some evidence of small-study effects, with Egger’s test suggesting potential bias favoring small studies with high sensitivity (*p* = 0.011). While trim-and-fill analysis indicated minimal impact on pooled estimates (adjusted sensitivity 95.2% vs. observed 95.8%), the possibility remains that unpublished studies with negative or null findings exist, particularly in the grey literature or as unpublished validation studies conducted by food industry laboratories or regulatory agencies. Comprehensive searches of regulatory databases, industry reports, and conference proceedings may identify additional unpublished data.

Finally, our analysis focused on diagnostic accuracy (sensitivity and specificity) as the primary outcomes, but did not systematically evaluate other important performance characteristics such as limit of detection, reproducibility, cost-effectiveness, labor requirements, or user-friendliness. A comprehensive comparison of direct culture versus enrichment should consider these practical factors alongside diagnostic accuracy, particularly for resource-limited settings where cost and simplicity may outweigh marginal gains in sensitivity or specificity.

We propose implementing a nuanced, risk-based approach in which method selection is guided by expected contamination levels and specific sample matrix characteristics. Direct culture (Procedure C) should be prioritized for high-load matrices—such as fresh poultry or cecal contents—to mitigate the profound polymyxin B-driven recovery bias that disproportionately suppresses *C. jejuni* during the enrichment phase. Conversely, selective enrichment remains indispensable for low-load or stressed samples, including frozen or processed products, where the resuscitation of sublethally injured cells is required for accurate detection.

Furthermore, in high-priority surveillance programs or regions with a high prevalence of ESBL-producing *E. coli*, a hybrid strategy should be considered. This approach may involve the routine use of tazobactam-supplemented mCCDA (T-mCCDA), which has been shown to enhance specificity by eliminating interference from background microflora without adversely affecting *Campylobacter* recovery. By combining direct culture for rapid, species-neutral quantification with enrichment for maximum sensitivity, food safety authorities can ensure more comprehensive pathogen surveillance and improved public health outcomes.

## 5. Conclusions

This systematic review and meta-analysis demonstrate that direct culture on mCCDA provides high diagnostic accuracy, yielding a pooled sensitivity of 99.1% and specificity of 97.9%, while significantly reducing turnaround times to 48 h. A critical finding is the identification of a profound species-specific recovery bias within current enrichment protocols. Specifically, the use of Bolton broth results in a 59.4 percentage-point lower recovery rate for *C. jejuni* than for *C. coli*, primarily due to the selective pressure of polymyxin B. Because *C. jejuni* accounts for approximately 90% of global human infections, this bias indicates that reliance on enrichment alone may lead to a systematic underestimation of the most clinically significant species in food products.

To address this diagnostic challenge, we propose a situational, risk-based approach for method selection that functions within the established ISO 10272-1:2017 framework. We recommend prioritizing direct culture (Procedure C) for high-contamination matrices, such as fresh poultry, to ensure the unbiased recovery of *C. jejuni* populations. While selective enrichment remains essential for resuscitating pathogens in low-load or stressed samples, adopting these situational refinements can significantly improve the accuracy of epidemiological data and food safety surveillance. Further large-scale validation is warranted to confirm these diagnostic advantages across diverse food matrices.

## Figures and Tables

**Table 1:** Characteristics of the 10 studies (43 comparisons) included in the meta-analysis of culture-based *Campylobacter* detection methods in food products.

Study [References]	Country	Sample Size	Comp.	Food Matrix	Enrichment Broth	Contam.
Biesta-Peters et al. (2018) [54]	Netherlands	2560	10	Frozen spinach; Minced meat; Raw milk; Chicken skin; Broiler caecal	Bolton broth; Preston broth; Direct plating	Artificial
Oliveira et al. (2024) [55]	Portugal	50	4	Fresh raw broiler meat; Fresh raw ground pork	Bolton/Preston broth	Artificial
Borck et al. (2002) [56]	Denmark	266	4	Turkey faecal and environmental samples	CEB and Preston broth	Natural
Chon et al. (2016) [57]	South Korea	240	2	Chicken carcass rinse	Buffered peptone water + 2x Bolton broth	Natural
Rodgers et al. (2016) [58]	UK	508	4	Chicken carcass rinse	Direct Culture (mCCDA); Preston broth; Bolton broth	Natural
Andritsos et al. (2020) [59]	Greece	349	8	Chicken meat	Bolton broth	Natural
Ben Bari et al. (2020) [60]	France	125	6	Poultry meat	Bolton	Artificial
Gonzales et al. (2022) [61]	United States	60	3	Raw ground chicken; Chicken carcass rinsate; Turkey carcass sponge	Blood-free Bolton broth	Artificial
Bailey et al. (2008) [62]	United States	398	1	Broiler carcass rinse	Bolton broth with 5% lysed horse blood	Natural
Debretsion et al. (2009) [63]	United States	43	1	Retail chicken	Bolton broth	Natural

Abbreviations: Comp. = Comparisons; Contam. = Contamination type; Sens. = Sensitivity; Spec. = Specificity; NR = Not reported. Notes: Sample sizes represent the total sample size across all comparisons within each study.

**Table 2:** Quality Assessment of Included Studies (QUADAS-2). Quality assessment of diagnostic accuracy studies using the QUADAS-2 tool. Risk of bias was assessed across four domains: patient selection, index test, reference standard, and flow and timing.

Study	Patient Selection	Index Test	Reference Standard	Flow & Timing	Overall Risk of Bias
Biesta-Peters et al. (2018) [54]	Low	Low	Low	Low	Low
Oliveira et al. (2024) [55]	Low	Low	Low	Low	Low
Borck et al. (2002) [56]	Unclear	Low	Low	Low	Unclear
Chon et al. (2016) [57]	Low	Low	Unclear	Low	Unclear
Rodgers et al. (2016) [58]	Low	Low	Low	Unclear	Unclear
Andritsos et al. (2020) [59]	Low	Low	Low	Low	Low
Ben Bari et al. (2020) [60]	Low	Low	Low	Low	Low
Gonzales et al. (2022) [61]	Low	Low	Low	Low	Low
Bailey et al. (2008) [62]	Low	Low	Low	Low	Low
Debretsion et al. (2009) [63]	Low	Low	Low	Low	Low

Summary Statistics: Overall Risk of Bias: 70% low risk (7/10 studies), 30% unclear risk (3/10 studies), 0% high risk - Overall Applicability Concerns: 100% low concern (10/10 studies). Notes: All studies showed low applicability concerns across all domains. Assessment categories: Low = low risk/concern; Unclear = unclear risk/concern; High = high risk/concern. QUADAS-2 domains assess: (1) patient selection for representativeness and bias, (2) index test conduct and interpretation, (3) reference standard appropriateness, and (4) flow and timing of tests.

**Table 3:** Individual Study Results—Diagnostic Accuracy Data. Diagnostic accuracy data for 43 method comparisons from 10 included studies showing true positives (TP), false positives (FP), true negatives (TN), false negatives (FN), sensitivity, and specificity with 95% confidence intervals.

	Study	Method	Matrix	n	TP	FP	TN	FN	Sensitivity (%)	95% CI	Specificity (%)	95% CI
1	Biesta-Peters 2018 [54]	Bolton	Spinach	256	205	3	45	3	98.6	(95.9–99.7)	93.8	(82.8–98.7)
2	Biesta-Peters 2018 [54]	Preston	Spinach	256	201	5	43	7	96.5	(93.3–98.6)	89.6	(77.3–96.5)
3	Biesta-Peters 2018 [54]	Bolton	Meat	256	203	4	44	5	97.6	(94.6–99.2)	91.7	(79.8–97.9)
4	Biesta-Peters 2018 [54]	Preston	Meat	256	198	6	42	10	95.2	(91.3–97.7)	87.5	(74.8–95.3)
5	Biesta-Peters 2018 [54]	Bolton	Milk	256	206	2	46	2	99.0	(96.6–99.9)	95.8	(85.8–99.5)
6	Biesta-Peters 2018 [54]	Preston	Milk	256	200	4	44	8	96.2	(92.7–98.3)	91.7	(79.8–97.9)
7	Biesta-Peters 2018 [54]	Bolton	Chicken	256	207	1	47	1	99.5	(97.3–100)	97.9	(88.9–99.9)
8	Biesta-Peters 2018 [54]	Preston	Chicken	256	202	3	45	6	97.1	(93.9–98.9)	93.8	(82.8–98.7)
9	Biesta-Peters 2018 [54]	Bolton	Caecal	256	208	0	48	0	100.0	(98.2–100)	100.0	(92.6–100)
10	Biesta-Peters 2018 [54]	Direct	Caecal	256	204	2	46	4	98.1	(95.2–99.5)	95.8	(85.8–99.5)
11	Oliveira 2024 [55]	Bolton-1	Broiler	13	9	01	2	1	90.0	(55.5–99.7)	66.7	(9.4–99.2)
12	Oliveira 2024 [55]	Preston-1	Broiler	13	8	2	1	2	80.0	(44.4–97.5)	33.3	(0.8–90.6)
13	Oliveira 2024 [55]	Bolton-2	Pork	12	8	1	2	1	88.9	(51.8–99.7)	66.7	(9.4–99.2)
14	Oliveira 2024 [55]	Preston-2	Pork	12	7	2	1	2	77.8	(40.0–97.2)	33.3	(0.8–90.6)
15	Borck 2002 [56]	CEB	Turkey	67	51	2	12	2	96.2	(86.8–99.5)	85.7	(57.2–98.2)
16	Borck 2002 [56]	Preston-1	Turkey	67	49	3	11	34	92.5	(81.8–97.9)	78.6	(49.2–95.3)
17	Borck 2002 [56]	Preston-2	Turkey	66	50	52	12	2	96.2	(86.8–99.5)	85.7	(57.2–98.2)
18	Borck 2002 [56]	Preston-3	Turkey	66	48	4	10	54	92.3	(81.5–97.9)	71.4	(41.9–91.6)
19	Chon 2016 [57]	BPW+Bolton-1	Carcass	120	96	2	20	2	98.0	(92.8–99.8)	90.9	(70.8–98.9)
20	Chon 2016 [57]	BPW+Bolton-2	Carcass	120	294	3	19	4	95.9	(89.9–98.9)	86.4	(65.1–97.1)
21	Rodgers 2016 [58]	Direct Culture	Carcass	127	125	0	2	0	100.0	(97.1–100)	100.0	(15.8–100)
22	Rodgers 2016 [58]	Preston	Carcass	127	118	0	2	7	94.4	(89.0–97.7)	100.0	(15.8–100)
23	Rodgers 2016 [58]	Bolton (*C.j*)	Carcass	127	110	0	2	15	88.0	(81.1–93.1)	100.0	(15.8–100)
24	Rodgers 2016 [58]	Bolton (*C.c*)	Carcass	127	122	0	2	3	97.6	(93.2–99.5)	100.0	(15.8–100)
25	Andritsos 2020 [59]	Bolton-1	Chicken	44	35	1	7	1	97.2	(85.5–99.9)	87.5	(47.3–99.7)
26	Andritsos 2020 [59]	Bolton-2	Chicken	44	34	1	8	2	94.4	(81.3–99.3)	88.9	(51.8–99.7)
27	Andritsos 2020 [59]	Bolton-3	Chicken	44	36	0	8	0	100.0	(90.3–100)	100.0	(63.1–100)
28	Andritsos 2020 [59]	Bolton-4	Chicken	43	33	2	6	2	94.3	(80.8–99.3)	75.0	(34.9–96.8)
29	Andritsos 2020 [59]	Bolton-5	Chicken	44	35	1	7	1	97.2	(85.5–99.9)	87.5	(47.3–99.7)
30	Andritsos 2020 [59]	Bolton-6	Chicken	43	34	1	7	1	97.1	(85.1–99.9)	87.5	(47.3–99.7)
31	Andritsos 2020 [59]	Bolton-7	Chicken	44	36	0	8	0	100.0	(90.3–100)	100.0	(63.1–100)
32	Andritsos 2020 [59]	Bolton-8	Chicken	43	33	2	7	1	97.1	(84.7–99.9)	77.8	(40.0–97.2)
33	Ben Bari 2020 [60]	Bolton-1	Poultry	21	14	1	5	1	93.3	(68.1–99.8)	83.3	(35.9–99.6)
34	Ben Bari 2020 [60]	Bolton-2	Poultry	21	16	0	6	0	100.0	(78.2–100)	100.0	(54.1–100)
35	Ben Bari 2020 [60]	Bolton-3	Poultry	21	13	2	4	2	86.7	(59.5–98.3)	66.7	(22.3–95.7)
36	Ben Bari 2020 [60]	Bolton-4	Poultry	21	14	1	5	1	93.3	(68.1–99.8)	83.3.7	(35.9–99.6)
37	Ben Bari 2020 [60]	Bolton-5	Poultry	20	13	1	5	1	92.9	(66.1–99.8)	83.3	(35.9–99.6)
38	Ben Bari 2020 [60]	Bolton-6	Poultry	21	15	0	6	0	100.0	(78.2–100)	100.0	(54.1–100)
39	Gonzales 2022 [61]	Blood-free-1	Chicken	20	15	1	3	1	93.8	(69.8–99.8)	75.0	(19.4–99.4)
40	Gonzales 2022 [61]	Blood-free-2	Carcass	20	16	0	4	0	100.0	(79.4–100)	100.0	(39.8–100)
41	Gonzales 2022 [61]	Blood-free-3	Turkey	20	14	1	4	1	93.3	(68.1–99.8)	80.0	(28.4–99.5)
42	Bailey 2008 [62]	Bolton+blood	Carcass	398	318	4	72	4	98.8	(96.9–99.7)	94.7	(87.1–98.5)
43	Debretsion 2009 [63]	Bolton	Chicken	43	33	2	6	2	94.3	(80.8–99.3)	75.0	(34.9–96.8)

Abbreviations: ID = Comparison identifier; n = Sample size; TP = True positives; FP = False positives; TN = True negatives; FN = False negatives; CI = Confidence interval (Wilson score method). Notes: Confidence intervals calculated using Wilson score method. Total comparisons: 43 from 10 unique studies. Sensitivity range: 77.8–100%. Specificity range: 33.3–100%. Data represents diagnostic accuracy against validated reference standards.

**Table 4:** Pooled Diagnostic Accuracy Estimates. Pooled sensitivity and specificity estimates from random-effects meta-analysis of 43 method comparisons from 10 studies. Estimates calculated using DerSimonian-Laird method with Wilson score confidence intervals.

Analysis	Comparisons (n)	Samples (n)	Pooled Sensitivity (%)	95% CI	I^2^ (%)	Pooled Specificity (%)	95% CI	I^2^ (%)
Overall (All Methods)	43	4599	95.8	(93.6–97.4)	72.3	90.2	(86.8–92.9)	68.7
By Detection Method:								
Direct Culture	2	383	99.1	(97.4–99.8)	0.0	97.9	(91.5–99.7)	0.0
Bolton Broth	31	3104	96.2	(94.1–97.7)	65.4	89.5	(85.7–92.5)	71.2
Preston Broth	8	1024	93.7	(89.2–96.6)	58.9	87.3	(80.1–92.4)	62.3
CEB Broth	1	67	96.2	(86.8–99.5)	—	85.7	(57.2–98.2)	—
BPW + Bolton	2	240	97.0	(92.6–99.0)	0.0	88.6	(73.3–96.2)	0.0
By Food Matrix:								
Chicken/Poultry Meat	20	1189	95.4	(92.5–97.4)	68.2	88.7	(83.4–92.7)	65.4
Carcass Rinse	8	1032	96.8	(93.7–98.6)	52.1	92.3	(85.2–96.4)	48.7
Multiple Matrices	10	2560	97.8	(96.4–98.8)	45.3	91.8	(87.9–94.7)	52.6
Turkey/Environmental	4	266	94.3	(88.5–97.6)	0.0	80.4	(67.2–89.6)	0.0
By Contamination Type:	** **							
Natural Contamination	25	1736	95.9	(93.4–97.6)	69.8	91.2	(87.1–94.3)	62.4
Artificial Contamination	18	2863	95.7	(92.8–97.6)	76.1	89.1	(84.2–92.8)	74.3

Abbreviations: CI = Confidence interval; I^2^ = I-squared statistic (measure of heterogeneity); n = Number of comparisons or samples; — = Not applicable (single study). Notes: Pooled estimates calculated using DerSimonian-Laird random-effects model. I^2^ values: 0–40% = low heterogeneity, 40–75% = moderate heterogeneity, >75% = high heterogeneity. Between-subgroup heterogeneity was statistically significant for detection method (Q = 18.7, *p* = 0.002) but not for food matrix (Q = 6.4, *p* = 0.17) or contamination type (Q = 0.8, *p* = 0.37).

**Table 5:** Subgroup Analysis by Detection Method, Food Matrix, and Study Characteristics. Detailed subgroup analysis showing pooled sensitivity and specificity with heterogeneity statistics and between-group comparisons. Analysis performed using random-effects meta-regression.

Subgroup	n	Sensitivity (%)	95% CI	I^2^	Specificity (%)	95% CI	I^2^	Q-Test	*p*-Value
Detection Method									
Direct Culture (mCCDA)	2	99.1	(97.4–99.8)	0.0%	97.9	(91.5–99.7)	0.0%	18.7	0.002
Bolton Broth (all variants)	31	96.2	(94.1–97.7)	65.4%	89.5	(85.7–92.5)	71.2%		
Preston Broth	8	93.7	(89.2–96.6)	58.9%	87.3	(80.1–92.4)	62.3%		
Other Methods	2	96.6	(89.4–99.2)	0.0%	87.2	(72.6–95.3)	0.0%		
Food Matrix									
Chicken/Poultry Meat	20	95.4	(92.5–97.4)	68.2%	88.7	(83.4–92.7)	65.4%	6.4	0.17
Carcass Rinse/Wash	8	96.8	(93.7–98.6)	52.1%	92.3	(85.2–96.4)	48.7%		
Multiple Matrices	10	97.8	(96.4–98.8)	45.3%	91.8	(87.9–94.7)	52.6%		
Turkey/Environmental	4	94.3	(88.5–97.6)	0.0%	80.4	(67.2–89.6)	0.0%		
Pork/Mixed	1	83.5	(62.7–94.8)	—	50.0	(18.7–81.3)	—		
Contamination Type									
Natural Contamination	25	95.9	(93.4–97.6)	69.8%	91.2	(87.1–94.3)	62.4%	0.8	0.37
Artificial Contamination	18	95.7	(92.8–97.6)	76.1%	89.1	(84.2–92.8)	74.3%		
Sample Size									
Small (<50 samples)	10	91.8	(86.2–95.6)	54.2%	77.3	(66.8–85.4)	48.9%	12.3	0.006
Medium (50–200 samples)	23	96.4	(93.9–98.0)	62.7%	91.5	(87.4–94.6)	58.3%		
Large (>200 samples)	10	97.6	(95.8–98.8)	48.1%	92.8	(88.7–95.7)	52.4%		
Geographic Region									
Europe	30	96.5	(94.4–98.0)	68.9%	90.8	(87.1–93.7)	65.2%	4.2	0.24
North America	9	95.8	(91.4–98.2)	58.7%	91.2	(84.3–95.6)	54.3%		
Asia	2	97.0	(92.6–99.0)	0.0%	88.6	(73.3–96.2)	0.0%		
Other	2	89.2	(77.8–95.7)	0.0%	72.5	(52.8–86.9)	0.0%		

Abbreviations: n = Number of comparisons; CI = Confidence interval; I^2^ = I-squared statistic; Q-test = Cochran’s Q test for between-subgroup heterogeneity; — = Not applicable (single comparison). Notes: Significant between-subgroup heterogeneity (*p* < 0.05) identified for detection method and sample size but not for food matrix, contamination type, or geographic region. Meta-regression showed sample size as a significant predictor of sensitivity (coefficient = 0.024, *p* = 0.018) and specificity (coefficient = 0.031, *p* = 0.012).

**Table 6:** Positive and Negative Predictive Values at Different Prevalence Levels. Calculated positive predictive value (PPV) and negative predictive value (NPV) for overall pooled estimates and detection method subgroups across clinically relevant Campylobacter prevalence scenarios in food products.

Detection Method	Sensitivity (%)	Specificity (%)	PPV at Prevalence (%)			NPV at Prevalence (%)		
			10%	30%	50%	10%	30%	50%
Overall (All Methods)	95.8	90.2	52.3	80.7	90.7	99.5	98.0	95.7
Direct Culture (mCCDA)	99.1	97.9	84.3	95.0	98.0	99.9	99.7	99.4
Bolton Broth (General)	96.2	89.5	49.8	79.5	90.2	99.6	98.2	96.1
Preston Broth	93.7	87.3	43.1	74.4	87.7	99.3	97.1	93.8
Scenario Analysis: High-Risk vs. Low-Risk Samples								
High-Risk Samples (70% prevalence)								
Direct Culture	99.1	97.9	—	—	—	—	—	98.7
Bolton Broth	96.2	89.5	—	—	—	—	—	94.8
Preston Broth	93.7	87.3	—	—	—	—	—	91.2
Low-Risk Samples (5% prevalence)								
Direct Culture	99.1	97.9	71.2	—	—	99.9	—	—
Bolton Broth	96.2	89.5	32.5	—	—	99.8	—	—
Preston Broth	93.7	87.3	27.8	—	—	99.7	—	—

Abbreviations: PPV = Positive predictive value; NPV = Negative predictive value; — = Not clinically relevant for this prevalence scenario). Calculation Method: PPV = (Sensitivity × Prevalence)/[(Sensitivity × Prevalence) + ((1 − Specificity) × (1 − Prevalence))]. NPV = (Specificity × (1 − Prevalence))/[((1 − Sensitivity) × Prevalence) + (Specificity × (1 − Prevalence))]. Interpretation: Prevalence scenarios: 5% = low-risk retail products (e.g., frozen chicken); 10% = typical retail chicken meat; 30% = fresh poultry carcasses; 50% = high-contamination scenarios (e.g., caecal contents); 70% = very high-risk samples (e.g., positive flocks). Direct culture maintains >95% PPV even at 30% prevalence, while enrichment methods show lower PPV at low prevalence (<50% at 10% prevalence). All methods maintain excellent NPV (>99%) at low prevalence, making them suitable for ruling out contamination. Clinical Significance: At typical retail prevalence (10–30%), direct culture offers superior positive predictive value (84–95%) compared to enrichment methods (43–80%), reducing false-positive rates and unnecessary product recalls. High NPV across all methods (>97%) confirms reliability for negative results.

## Data Availability

The original contributions presented in this study are included in the article/Supplementary Material. Further inquiries can be directed to the corresponding author.

## Supplementary Materials

The following supporting information can be downloaded at: https://www.mdpi.com/article/10.3390/vetsci13050415/s1, Figure S1: Standard funnel plot showing sensitivity estimates from 43 method comparisons, plotted against their standard errors; Figure S2: Contour-enhanced funnel plot with shaded regions indicating statistical significance levels; Figure S3: Regression plot showing precision (1/SE) vs. standardized effect; Figure S4: Standard funnel plot for specificity estimates; Figure S5: Contour-enhanced funnel plot for specificity with significance regions; Regression plot for specificity showing precision vs. standardized effect; Figure S6: Regression plot for specificity showing precision vs. standardized effect; Table S1: Complete search strategies for all databases; Table S2: Excluded studies with reasons for exclusion (n = 58); Table S3: Detailed study characteristics and methodology; Table S4: Complete diagnostic accuracy data with calculations; Table S5: Sensitivity Analysis Results; Table S6: Meta-Regression Coefficients; Table S7: Leave-One-Out Analysis Results; Table S8: Subgroup Analysis Detailed Statistics. File S1: Supplementary methods and results; File S2: PRISMA checklist.

## References

[1] Kirk M.D., Pires S.M., Black R.E., Caipo M., Crump J.A., Devleesschauwer B., Döpfer D., Fazil A., Fischer-Walker C.L., Hald T. (2015). World Health Organization estimates of the global and regional disease burden of 22 foodborne bacterial, protozoal, and viral diseases, 2010: A Data Synthesis. PLoS Med..

[2] Kaakoush N.O., Castaño-Rodríguez N., Mitchell H.M., Man S.M. (2015). Global epidemiology of *Campylobacter* infection. Clin. Microbiol. Rev..

[3] European Food Safety Authority (EFSA), European Centre for Disease Prevention and Control (ECDC) (2024). The european union one health 2023 zoonoses report. EFSA J..

[4] Tack D.M., Ray L., Griffin P.M., Cieslak P.R., Dunn J., Rissman T., Jervis R., Lathrop S., Muse A., Duwell M. (2020). Preliminary incidence and trends of infections with pathogens transmitted commonly through food- foodborne diseases active surveillance network, 10 U.S. sites, 2016–2019. MMWR Morb. Mortal. Wkly. Rep..

[5] Skarp C.P.A., Hänninen M.-L., Rautelin H.I.K. (2016). Campylobacteriosis: The role of poultry meat. Clin. Microbiol. Infect..

[6] Suzuki H., Yamamoto S. (2009). *Campylobacter* contamination in retail poultry meats and by-products in the world: A literature survey. J. Vet. Med. Sci..

[7] Lévesque S., Fournier E., Carrier N., Frost E., Arbeit R.D., Michaud S. (2013). Campylobacteriosis in urban versus rural areas: A case-case study integrated with molecular typing to validate risk factors and to attribute sources of infection. PLoS ONE.

[8] Hald B., Wedderkopp A., Madsen M. (2000). Thermophilic *Campylobacter* spp. in danish broiler production: A cross-sectional survey and a retrospective analysis of risk factors for occurrence in broiler flocks. Avian Pathol..

[9] Black R.E., Levine M.M., Clements M.L., Hughes T.P., Blaser M.J. (1988). Experimental *Campylobacter jejuni* infection in humans. J. Infect Dis..

[10] Nauta M., Hill A., Rosenquist H., Brynestad S., Fetsch A., Van Der Logt P., Fazil A., Christensen B., Katsma E., Borck B. (2009). A comparison of risk assessments on *Campylobacter* in broiler meat. Int. J. Food Microbiol..

[11] Silva J., Leite D., Fernandes M., Mena C., Gibbs P.A., Teixeira P. (2011). *Campylobacter* spp. as a foodborne pathogen: A review. Front. Microbiol..

[12] Humphrey T.J. (1989). An appraisal of the efficacy of pre-enrichment for the isolation of *Campylobacter jejuni* from water and food. J. Appl. Bacteriol..

[13] Epps S., Harvey R., Hume M., Phillips T., Anderson R., Nisbet D. (2013). Foodborne *Campylobacter*: Infections, metabolism, pathogenesis and reservoirs. Int. J. Environ. Res. Public Health.

[14] Jacobs-Reitsma W.F., Bolder N.M., Mulder R.W.A.W. (1994). Cecal carriage of *Campylobacter* and *Salmonella* in Dutch broiler flocks at slaughter: A one-year study. Poult. Sci..

[15] Rosenquist H., Nielsen N.L., Sommer H.M., Nørrung B., Christensen B.B. (2003). Quantitative risk assessment of human campylobacteriosis associated with thermophilic *Campylobacter* species in chickens. Int. J. Food Microbiol..

[16] Parkhill J., Wren B.W., Mungall K., Ketley J.M., Churcher C., Basham D., Chillingworth T., Davies R.M., Feltwell T., Holroyd S. (2000). The genome sequence of the food-Borne pathogen *Campylobacter jejuni* reveals hypervariable sequences. Nature.

[17] Alter T., Scherer K. (2006). Stress response of *Campylobacter* spp. and its role in food processing. J. Vet. Med. B Infect. Dis. Vet. Public Health.

[18] Oliver J.D. (2010). Recent findings on the viable but nonculturable state in pathogenic bacteria. FEMS Microbiol. Rev..

[19] Baffone W., Casaroli A., Citterio B., Pierfelici L., Campana R., Vittoria E., Guaglianone E., Donelli G. (2006). *Campylobacter jejuni* loss of culturability in aqueous microcosms and ability to resuscitate in a ouse model. Int. J. Food Microbiol..

[20] Rollins D.M., Colwell R.R. (1986). Viable but nonculturable stage of *Campylobacter jejuni* and its role in survival in the natural aquatic environment. Appl. Environ. Microbiol..

[21] (2017). Microbiology of the Food Chain—Horizontal Method for Detection and Enumeration of Campylobacter spp.—Part 1: Detection Method.

[22] Hunt J.M., Abeyta C., Tran T. (2001). Campylobacter. Bacteriological Analytical Manual.

[23] Corry J.E., Post D.E., Colin P., Laisney M.J. (1995). Culture media for the isolation of *Campylobacters*. Int. J. Food Microbiol..

[24] On S.L.W. (2013). Isolation, Identification and subtyping of *Campylobacter*: Where to from here?. J. Microbiol. Methods.

[25] Fitzgerald C., Patrick M., Gonzalez A., Akin J., Polage C.R., Wymore K., Gillim-Ross L., Xavier K., Sadlowski J., Monahan J. (2016). Multicenter evaluation of clinical diagnostic methods for detection and isolation of *Campylobacter* spp. from stool. J. Clin. Microbiol..

[26] Humphrey T., O’Brien S., Madsen M. (2007). *Campylobacters* as zoonotic pathogens: A food roduction perspective. Int. J. Food Microbiol..

[27] Steele T.W., McDermott S.N. (1984). The use of membrane filters applied directly to the surface of agar plates for the isolation of *Campylobacter jejuni* from feces. Pathology.

[28] Bolton F.J., Hutchinson D.N., Parker G. (1988). Reassessment of selective agars and filtration techniques for isolation of *Campylobacter* species from faeces. Eur. J. Clin. Microbiol. Infect. Dis..

[29] Bolton F.J., Coates D., Hutchinson D.N., Godfree A.F. (1987). A study of thermophilic *Campylobacters* in a river system. J. Appl. Bacteriol..

[30] Baylis C.L., MacPhee S., Martin K.W., Humphrey T.J., Betts R.P. (2000). Comparison of three enrichment media for the isolation of *Campylobacter* spp. from foods. J. Appl. Microbiol..

[31] Brueggemann-Schwarze S., Preuß S., Goenaga J.C., Buhler C., Heise J., Stingl K. (2025). Quantitative comparison of thermotolerant *Campylobacter* spp. growth in preston broth with and without growth supplement. Int. J. Food Microbiol..

[32] Line J.E. (2001). Development of a selective differential agar for isolation and enumeration of *Campylobacter* spp.. J. Food Prot..

[33] Hutchinson D.N., Bolton F.J. (1984). Improved blood free selective medium for the isolation of *Campylobacter jejuni* from faecal specimens. J. Clin. Pathol..

[34] Aspinall S.T., Wareing D.R., Hayward P.G., Hutchinson D.N. (1993). Selective medium for thermophilic *Campylobacters* including *Campylobacter upsaliensis*. J. Clin. Pathol..

[35] Ghosh K., Logno T.A., Das T., Dhar P.K., Blake D., Fournie G., Tomley F., Stabler R.A., Lehri B., Biswas P.K. (2025). Optimizing ISO standard microbiological techniques for isolating *Campylobacter* from poultry samples amidst challenges from extended spectrum beta lactamase producing *Escherichia coli*. PLoS ONE.

[36] He Y., Capobianco J., Armstrong C.M., Chen C.-Y., Counihan K., Lee J., Reed S., Tilman S. (2024). Detection and isolation of *Campylobacter* spp. from raw meat. J. Vis. Exp..

[37] Corry J.E.L., Atabay H.I. (2001). Poultry as a source of *Campylobacter* and related organisms. J. Appl. Microbiol..

[38] Moore J.E., Corcoran D., Dooley J.S.G., Fanning S., Lucey B., Matsuda M., McDowell D.A., MéGraud F., Millar B.C., O’Mahony R. (2005). Campylobacter. Vet. Res..

[39] Engberg J., On S.L.W., Harrington C.S., Gerner-Smidt P. (2000). Prevalence of *Campylobacter*, *Arcobacter*, *Helicobacter*, and *Sutterella* spp. in human fecal samples as estimated by a reevaluation of isolation methods for *Campylobacters*. J. Clin. Microbiol..

[40] Beumer R.R., De Vries J., Rombouts F.M. (1992). *Campylobacter jejuni* non-culturable coccoid cells. Int. J. Food Microbiol..

[41] Page M.J., McKenzie J.E., Bossuyt P.M., Boutron I., Hoffmann T.C., Mulrow C.D., Shamseer L., Tetzlaff J.M., Akl E.A., Brennan S.E. (2021). The PRISMA 2020 statement: An updated guideline for reporting systematic reviews. BMJ.

[42] Deeks J.J., Bossuyt P.M., Leeflang M.M., Takwoingi Y. (2010). Cochrane Handbook for Systematic Reviews of Diagnostic Test Accuracy Version 1.0.

[43] Bossuyt P.M., Reitsma J.B., Bruns D.E., Gatsonis C.A., Glasziou P.P., Irwig L., Lijmer J.G., Moher D., Rennie D., de Vet H.C.W. (2015). STARD 2015: An updated list of essential items for reporting diagnostic accuracy studies. BMJ.

[44] Whiting P.F., Rutjes A.W.S., Westwood M.E., Mallett S., Deeks J.J., Reitsma J.B., Leeflang M.M.G., Sterne J.A.C., Bossuyt P.M.M., QUADAS-2 Group (2011). QUADAS-2: A revised tool for the quality assessment of diagnostic accuracy studies. Ann. Intern. Med..

[45] Wilson E.B. (1927). Probable inference, the law of succession, and statistical inference. J. Am. Stat. Assoc..

[46] Newcombe R.G. (1998). Two-sided confidence intervals for the single proportion: Comparison of seven methods. Stat. Med..

[47] DerSimonian R., Laird N. (1986). Meta-analysis in clinical trials. Control. Clin. Trials.

[48] Higgins J.P., Thompson S.G. (2002). Quantifying heterogeneity in a meta-analysis. Stat. Med..

[49] Deeks J.J., Higgins J.P., Altman D.G., Higgins J.P., Thomas J., Chandler J., Cumpston M., Li T., Page M., Welch V., Flemyng E. (2021). Chapter 10: Analysing data and undertaking meta-analyses. Cochrane Handbook for Systematic Reviews of Interventions (Version 6.2).

[50] Knapp G., Hartung J. (2003). Improved tests for a random effects meta-regression with a single covariate. Stat. Med..

[51] Egger M., Smith G.D., Schneider M., Minder C. (1997). Bias in meta-analysis detected by a simple, graphical test. BMJ.

[52] Begg C.B., Mazumdar M. (1994). Operating characteristics of a rank correlation test for publication bias. Biometrics.

[53] Duval S., Tweedie R. (2000). Trim and fill: A simple funnel-plot-based method of testing and adjusting for publication bias in meta-analysis. Biometrics.

[54] Biesta-Peters E.G., Jongenburger I., de Boer E., Jacobs-Reitsma W.F. (2018). Validation by interlaboratory trials of EN ISO 10272 - Microbiology of the food chain - Horizontal method for detection and enumeration of Cam-pylobacter spp. - Part 1: Detection method. Int. J. Food Microbiol..

[55] Oliveira R., Barbosa A., Sousa M., Azevedo N.F., Cerqueira L., Almeida C. (2024). Using peptide nucleic acid fluo-rescence in situ hybridization (PNA-FISH) to detect Campylobacter spp. in food samples. LWT-Food Sci.Technol.

[56] Borck B., Stryhn H., Ersboll A.K., Pedersen K. (2002). Thermophilic Campylobacter spp. in Turkey samples: Evaluation of two automated enzyme immunoassays and conventional microbiological techniques. J. Appl. Microbi-ol..

[57] Chon J.-W., Kim Y.-J., Kim H.-S., Kim D.-H., Jeong D.K., Seo K.-H. (2016). Evaluation of tazobac-tam-supplemented, modified charcoal-cefoperazone-deoxycholate agar for qualitative detection of Campylo-bacter from chicken carcass rinse. Foodborne Pathog. Dis..

[58] Rodgers J.D., Simpkin E., Lee R., Clifton‐Hadley F.A., Vidal A.B. (2017). Sensitivity of direct culture, enrichment and PCR for detection of Campylobacter jejuni and C. coli in broiler flocks at slaughter. Zoonoses Public Health.

[59] Andritsos N.D., Tzimotoudis N., Mataragas M. (2020). Estimating the performance of four culture edia used for enumeration and detection of Campylobacter species in chicken meat. LWT-Food Sci. Technol..

[60] Bari S.B., Dangerville M., Neve R.L., Boubetra A., Tirard A. (2020). Proficiency-testing scheme for detection and enumeration of Campylobacter in poultry meat. Acta Sci. Microbiol..

[61] Gonzalez V., Juck G., Sutzko M., Muldoon M.T. (2022). Validation of rapidchek®Campylobacter test system for the detection of C.jejuni, C. coli, and C. lari in poultry samples: AOAC performance tested methodSM 052201. J. AOAC Int..

[62] Bailey J.S., Lyon B.G., Lyon C.E., Windham W.R. (2000). The microbiological profile of chilled and frozen chicken. J. Food Prot..

[63] Debretsion A., Habtemariam T., Wilson S., Tameru B., Wesley I.V., Yehualaeshet T. (2009). Comparative assess-ment of standard culture and real‐time polymerase chain reaction to detect Campylobacter jejuni in retail chicken samples. J. Food Saf..

[64] Bolton F.J., Coates D., Hutchinson D.N. (1984). The ability of *Campylobacter* media incorporating blood or blood products to neutralise the effect of inhibitory substances in faeces. J. Appl. Bacteriol..

[65] Humphrey T.J., Henley A., Lanning D.G. (1993). The colonization of broiler chickens with *Campylobacter jejuni*: Some epidemiological investigations. Epidemiol. Infect..

[66] Hänninen M.L., Haajanen H., Pummi T., Wermundsen K., Katila M.L., Sarkkinen H., Miettinen I., Rautelin H. (2003). Detection and typing of *Campylobacter jejuni* and Campylobacter coli and analysis of indicator organisms in three waterborne outbreaks in Finland. Appl. Environ. Microbiol..

[67] Karmali M.A., Simor A.E., Roscoe M., Fleming P.C., Smith S.S., Lane J. (1986). Evaluation of a blood-free, charcoal-based, selective medium for the isolation of *Campylobacter* organisms from feces. J. Clin. Microbiol..

[68] Stern N.J., Robach M.C. (2003). Enumeration of *Campylobacter* spp. in broiler feces and in corresponding processed carcasses. J. Food Prot..

[69] Newell D.G., Fearnley C. (2003). Sources of *Campylobacter* colonization in broiler chickens. Appl. Environ. Microbiol..

[70] Murphy C., Carroll C., Jordan K.N. (2006). Environmental survival mechanisms of the foodborne pathogen *Campylobacter jejuni*. J. Appl. Microbiol..

[71] Berndtson E., Danielsson-Tham M.L., Engvall A. (1996). *Campylobacter* incidence on a chicken farm and the spread of *Campylobacter* during the slaughter process. Int. J. Food Microbiol..

[72] Bronowski C., James C.E., Winstanley C. (2014). Role of environmental survival in transmission of *Campylobacter jejuni*. FEMS Microbiol. Lett..

[73] Tholozan J.L., Cappelier J.M., Tissier J.P., Delattre G., Federighi M. (1999). Physiological characterization of viable-but-nonculturable *Campylobacter jejuni* cells. Appl. Environ. Microbiol..

[74] Cappelier J.M., Minet J., Magras C., Colwell R.R., Federighi M. (1999). Recovery in embryonated eggs of viable but nonculturable *Campylobacter jejuni* cells and maintenance of ability to adhere to HeLa cells after resuscitation. Appl. Environ. Microbiol..

[75] Bolton F.J., Robertson L. (1982). A selective medium for isolating *Campylobacter jejuni*/*coli*. J. Clin. Pathol..

[76] Velkov T., Roberts K.D., Nation R.L., Thompson P.E., Li J. (2013). Pharmacology of polymyxins: New insights into an ‘old’ class of antibiotics. Future Microbiol..

[77] Trimble M.J., Mlynárčik P., Kolář M., Hancock R.E.W. (2016). Polymyxin: Alternative mechanisms of action and resistance. Cold Spring Harb. Perspect. Med..

[78] Landman D., Georgescu C., Martin D.A., Quale J. (2008). Polymyxins revisited. Clin. Microbiol. Rev..

[79] Luangtongkum T., Jeon B., Han J., Plummer P., Logue C.M., Zhang Q. (2009). Antibiotic resistance in *Campylobacter*: Emergence, transmission and persistence. Futur. Microbiol..

[80] Alfredson D.A., Korolik V. (2007). Antibiotic resistance and resistance mechanisms in *Campylobacter jejuni* and *Campylobacter coli*. FEMS Microbiol. Lett..

[81] Ge B., Wang F., Sjölund-Karlsson M., McDermott P.F. (2013). Antimicrobial resistance in *Campylobacter*: Susceptibility testing methods and resistance trends. J. Microbiol. Methods.

[82] Engberg J., Aarestrup F.M., Taylor D.E., Gerner-Smidt P., Nachamkin I. (2001). Quinolone and macrolide re-sistance in Campylobacter jejuni and C. coli: Resistance mechanisms and trends in human isolates. Emerging Infectious Diseases.

[83] Guerry P., Szymanski C.M., Prendergast M.M., Hickey T.E., Ewing C.P., Pattarini D.L., Moran A.P. (2002). Phase variation of *Campylobacter jejuni* 81-176 lipooligosaccharide affects ganglioside mimicry and invasiveness in vitro. Infect. Immun..

[84] Szymanski C.M., King M., Haardt M., Armstrong G.D. (1995). *Campylobacter jejuni* motility and invasion of Caco-2 cells. Infect. Immun..

[85] Karlyshev A.V., Linton D., Gregson N.A., Wren B.W. (2002). A novel paralogous gene family involved in phase-variable flagella-mediated motility in *Campylobacter jejuni*. Microbiology.

[86] Monteiro M.A., Baqar S., Hall E.R., Chen Y.H., Porter C.K., Bentley B.E., Applebee L., Guerry P. (2009). Capsule polysaccharide conjugate vaccine against diarrheal disease caused by *Campylobacter jejuni*. Infect. Immun..

[87] Louwen R., Heikema A., van Belkum A., Ott A., Gilbert M., Ang W., Endtz H.P., Bergman M.P., Nieuwenhuis E.E. (2008). The sialylated lipooligosaccharide outer core in *Campylobacter jejuni* is an important determinant for epithelial cell invasion. Infect. Immun..

[88] Cullen T.W., Schofield W.B., Barry N.A., Putnam E.E., Rundell E.A., Trent M.S., Degnan P.H., Booth C.J., Yu H., Goodman A.L. (2015). Antimicrobial peptide resistance mediates resilience of prominent gut commensals during inflammation. Science.

[89] Raetz C.R.H., Reynolds C.M., Trent M.S., Bishop R.E. (2007). Lipid A modification systems in Gram-negative bacteria. Annu. Rev. Biochem..

[90] Needham B.D., Trent M.S. (2013). Fortifying the barrier: The impact of lipid A remodelling on bacterial pathogenesis. Nat. Rev. Microbiol..

[91] Szymanski C.M., Gaynor E.C. (2012). How a sugary bug gets through the day: Recent developments in understanding fundamental processes impacting Campylobacter jejuni pathogenesis. Gut Microbes.

[92] Karlyshev A.V., Champion O.L., Churcher C., Brisson J.R., Jarrell H.C., Gilbert M., Brochu D., St Michael F., Li J., Wakarchuk W.W. (2005). Analysis of Campylobacter jejuni capsular loci reveals multiple mechanisms for the generation of structural diversity and the ability to form complex heptoses. Mol. Microbiol..

[93] United States Department of Agriculture, Food Safety and Inspection Service (2018). Meat and Poultry Hazards and Controls Guide.

[94] Luber P., Wagner J., Hahn H., Bartelt E. (2006). Quantification of *Campylobacter* species cross-contamination during handling of contaminated fresh chicken parts in kitchens. Appl. Environ. Microbiol..

[95] Whyte R., Hudson J.A., Graham C. (2006). *Campylobacter* in chicken livers and their destruction by pan frying. Lett. Appl. Microbiol..

[96] Oyarzabal O.A., Battie C., Hui Y.H., Evranuz E.Ö., Ötleş M., Ötleş M. (2012). *Campylobacter* spp.: Detection and quantification. Handbook of Plant-Based Fermented Food and Beverage Technology.

[97] Birk T., Ingmer H., Andersen M.T., Jørgensen K., Brøndsted L. (2004). Chicken juice, a food-based model system suitable to study survival of *Campylobacter jejuni*. Lett. Appl. Microbiol..

[98] Fernández H., Pisón V. (1996). Isolation of thermotolerant species of *Campylobacter* from commercial chicken livers. Int. J. Food Microbiol..

