# Supplementary material for: Comparative Performance and Species-Specific Recovery Biases of Culture-Based Methods for Campylobacter Detection in Food Products: A Systematic Review and Meta-Analysis
Source: Vet Sci. 2026 Apr 23;13(5):415. doi: 10.3390/vetsci13050415 (PMC13211414; doi:10.3390/vetsci13050415)
Supplement: Supplementary file 1 [file vetsci-13-00415-s001.zip › S1 Supplementary Methods and Results.pdf]

SUPPLEMENTARY METHODS

SECTION S1. Search Strategy Development Process

Step 1: Preliminary Scoping Search (- Conducted exploratory searches in PubMed and Google Scholar - Identified 20 potentially relevant studies - Extracted key terms and MeSH headings - Consulted with information specialist

Step 2: MeSH Term Identification - Primary MeSH terms: - "Campylobacter" [MeSH Major Topic] - "Food Microbiology" [MeSH] - "Food Contamination" [MeSH] - "Sensitivity and Specificity" [MeSH] - "Diagnostic Techniques and Procedures" [MeSH]

Step 3: Keyword Development - Generated comprehensive keyword list through: - Analysis of titles/abstracts of relevant studies - Review of ISO 10272-1:2017 terminology - Consultation with food microbiology experts - Thesaurus expansion using PubMed's automatic term mapping

Step 4: Search String Pilot Testing - Tested 5 different search string variations - Evaluated precision (proportion of relevant records retrieved) - Evaluated recall (ability to retrieve known relevant studies) - Selected optimal balance between sensitivity and specificity of search

Step 5: Database-Specific Adaptation - Adapted search strategy for each database's syntax: - PubMed: MeSH terms + text words with Boolean operators - Google Scholar: Natural language query with proximity operators - SciSpace: Semantic search with AI-powered relevance ranking - Web of Science: Topic field searches with wildcards

Step 6: Peer Review of Search Strategy - Independent review by information specialist - Checklist-based evaluation using PRESS guidelines - Modifications based on feedback

Search Filters Applied

Date Filter: - Start date: January 1, 2000 (rationale: modern detection methods) - End date: January 15, 2026 (search execution date)

Language Filter: - English language only - Rationale: Resource constraints, most food microbiology literature published in English

Document Type Filter: - Original research articles only - Excluded: Reviews, editorials, letters, comments, conference abstracts

Species Filter: - *Campylobacter* species only - Excluded: Studies on other foodborne pathogens without *Campylobacter*-specific data

SECTION S2. Data Extraction Forms and Procedures

Data Extraction Form Structure

A standardized data extraction form was developed in Microsoft Excel with 37 data fields organized into 8 categories:

Category 1: Study Identification (5 fields) 1. First author surname 2. Publication year 3. Journal name 4. DOI 5. Country of study

Category 2: Study Design (6 fields) 6. Study design (cross-sectional, cohort, experimental) 7. Setting (laboratory, processing plant, retail) 8. Sampling method 9. Sample size calculation reported (yes/no) 10. Blinding procedures 11. Funding source

Category 3: Sample Characteristics (7 fields) 12. Food matrix (chicken, beef, pork, vegetables, etc.) 13. Sample type (raw, processed, retail, etc.) 14. Total sample size 15. Contamination type (natural, artificial) 16. Contamination level (CFU/g or CFU/sample) 17. Sample storage conditions 18. Time from collection to analysis

Category 4: Index Test Details (8 fields) 19. Enrichment broth type (Bolton, Preston, CEB, etc.) 20. Enrichment broth composition 21. Enrichment incubation time 22. Enrichment incubation temperature 23. Enrichment atmosphere 24. Selective agar type (mCCDA, CCDA, etc.) 25. Agar incubation time 26. Confirmation method (biochemical, PCR, MALDI-TOF, etc.)

Category 5: Reference Standard (4 fields) 27. Reference standard method 28. Reference standard composition (if composite) 29. Independence from index test (yes/no) 30. Validation status of reference standard

Category 6: Diagnostic Accuracy Data (4 fields) 31. True Positives (TP) 32. False Positives (FP) 33. True Negatives (TN) 34. False Negatives (FN)

Category 7: Calculated Outcomes (3 fields) 35. Sensitivity (%) with 95% CI 36. Specificity (%) with 95% CI 37. Diagnostic Odds Ratio

Data Extraction Procedures

Dual Independent Extraction: - Two reviewers (initials: [AA], [BB]) independently extracted data from each study - Extraction performed in parallel without consultation - Discrepancies identified through automated comparison in Excel - Third reviewer ([CC]) adjudicated unresolved discrepancies

Piloting Phase: - Data extraction form piloted on 5 studies - Modifications made to improve clarity and completeness - Inter-rater agreement assessed (Cohen’s kappa = 0.92)

Extraction Process: 1. Read full text of study 2. Complete data extraction form 3. Calculate diagnostic accuracy metrics using 2×2 table 4. Flag any missing data or ambiguities 5. Contact study authors if critical data missing

Author Contact: - 12 authors contacted for missing diagnostic accuracy data - Response rate: 5/12 (42%) - 2 studies excluded due to non-response and insufficient published data

Quality Checks: - Automated range checks (e.g., sensitivity ≤100%) - Logical consistency checks (e.g., TP + FN = total diseased) - Cross-validation of calculated vs. reported values

---

SECTION S3. Statistical Analysis Code

Software and Packages Used

Primary Analysis Software: - **Python 3.9.7** with libraries: - NumPy 1.21.2 (numerical computations) - SciPy 1.7.1 (statistical tests) - Pandas 1.3.3 (data manipulation) - Matplotlib 3.4.3 (visualization) - Seaborn 0.11.2 (statistical visualization)

Meta-Analysis Packages: - **metafor** (R package version 3.4-0) for meta-regression - **mada** (R package version 0.5.11) for bivariate meta-analysis - **meta** (R package version 5.2-0) for forest plots

Code Repository: - All analysis code available at: [GitHub repository URL to be added] - Reproducible analysis workflow using Jupyter Notebooks

Meta-Analysis Code (Python)

```
import numpy as np
from scipy import stats

def calculate_wilson_ci(successes, total, confidence=0.95):
    """
    Calculate Wilson score confidence interval for proportions.

    Parameters:
    -----------
    successes : int
        Number of successes (TP or TN)
    total : int
        Total sample size (TP+FN or TN+FP)
    confidence : float
        Confidence level (default 0.95 for 95% CI)

    Returns:
    --------
    tuple : (lower_bound, upper_bound)
    """
    if total == 0:
        return (0, 0)

    p = successes / total
    z = stats.norm.ppf((1 + confidence) / 2)

    denominator = 1 + z**2 / total
    center = (p + z**2 / (2 * total)) / denominator
    margin = z * np.sqrt((p * (1 - p) / total + z**2 / (4 * total**2))) / denominator
```

```python
    lower = max(0, center - margin)
    upper = min(1, center + margin)

    return (lower * 100, upper * 100)

def dersimonian_laird_pooling(estimates, variances):
    """
    DerSimonian-Laird random-effects meta-analysis.

    Parameters:
    -----------
    estimates : array
        Study-specific estimates (sensitivity or specificity)
    variances : array
        Study-specific variances

    Returns:
    --------
    dict : pooled_estimate, pooled_variance, tau_squared, I_squared
    """
    # Calculate fixed-effects weights
    weights = 1 / variances

    # Fixed-effects pooled estimate
    pooled_fixed = np.sum(weights * estimates) / np.sum(weights)

    # Q statistic
    Q = np.sum(weights * (estimates - pooled_fixed)**2)
    df = len(estimates) - 1

    # Between-study variance (tau-squared)
    C = np.sum(weights) - np.sum(weights**2) / np.sum(weights)
    tau_squared = max(0, (Q - df) / C)

    # Random-effects weights
    weights_random = 1 / (variances + tau_squared)

    # Random-effects pooled estimate
    pooled_random = np.sum(weights_random * estimates) / np.sum(weights_random)
    pooled_variance = 1 / np.sum(weights_random)

    # I-squared statistic
    I_squared = max(0, 100 * (Q - df) / Q) if Q > 0 else 0

    return {
        'pooled_estimate': pooled_random,
        'pooled_variance': pooled_variance,
        'tau_squared': tau_squared,
```

```
    'I_squared': I_squared,
    'Q': Q,
    'p_value': 1 - stats.chi2.cdf(Q, df)
  }

# Example usage
sensitivity_values = np.array([97.2, 94.4, 100.0, ...])  # All 43 values
se_sensitivity = np.array([3.7, 4.6, 2.5, ...])  # Standard errors
variances = se_sensitivity ** 2

results = dersimonian_laird_pooling(sensitivity_values, variances)
print(f"Pooled Sensitivity: {results['pooled_estimate']:.1f}%")
print(f"I²: {results['I_squared']:.1f}%")
```

Egger's Regression Test Code

```
from scipy.stats import linregress

def eggers_test(estimates, standard_errors, pooled_estimate):
  """
  Egger's regression test for funnel plot asymmetry.

  Parameters:
  -----------
  estimates : array
    Study-specific estimates
  standard_errors : array
    Study-specific standard errors
  pooled_estimate : float
    Pooled estimate from meta-analysis

  Returns:
  --------
  dict : intercept, slope, p_value, interpretation
  """
  # Calculate precision (1/SE)
  precision = 1 / standard_errors

  # Calculate standardized effect
  standardized_effect = (estimates - pooled_estimate) / standard_errors

  # Remove infinite or NaN values
  valid = np.isfinite(precision) & np.isfinite(standardized_effect)
  precision_clean = precision[valid]
  standardized_effect_clean = standardized_effect[valid]

  # Linear regression
  slope, intercept, r_value, p_value, std_err = linregress(
    precision_clean, standardized_effect_clean
  )
```

```
    # Interpretation
    if p_value < 0.05:
        interpretation = "Significant asymmetry detected (p < 0.05)"
    else:
        interpretation = "No significant asymmetry (p ≥ 0.05)"

    return {
        'intercept': intercept,
        'slope': slope,
        'std_err': std_err,
        'p_value': p_value,
        'r_squared': r_value**2,
        'interpretation': interpretation
    }

# Example usage
egger_results = eggers_test(sensitivity_values, se_sensitivity, 95.8)
print(f"Egger's Test: Intercept = {egger_results['intercept']:.3f}, p = {egger_results['p_value']:.4f}")
```

SECTION S4. Quality Assessment Procedures

QUADAS-2 Tool Application

Domain 1: Patient Selection (Risk of Bias)

Signaling Questions: 1. Was a consecutive or random sample of patients enrolled? 2. Was a case-control design avoided? 3. Did the study avoid inappropriate exclusions?

Judgment Criteria: - **Low Risk:** All signaling questions answered “Yes” - **High Risk:** Any signaling question answered “No” - **Unclear Risk:** Insufficient information to judge

Domain 1: Patient Selection (Applicability Concerns)

Signaling Question: - Are there concerns that the included patients do not match the review question?

Judgment Criteria: - **Low Concern:** Food samples representative of target population - **High Concern:** Highly selected or unrepresentative samples - **Unclear Concern:** Insufficient information

Domain 2: Index Test (Risk of Bias)

Signaling Questions: 1. Were the index test results interpreted without knowledge of reference standard results? 2. If a threshold was used, was it pre-specified?

Judgment Criteria: - **Low Risk:** Both questions answered “Yes” - **High Risk:** Either question answered “No” - **Unclear Risk:** Insufficient information

Domain 2: Index Test (Applicability Concerns)

Signaling Question: - Are there concerns that the index test, its conduct, or interpretation differ from the review question?

Judgment Criteria: - **Low Concern:** Standard culture methods as per ISO protocols - **High Concern:** Modified or non-standard methods - **Unclear Concern:** Insufficient detail on methodology

Domain 3: Reference Standard (Risk of Bias)

Signaling Questions: 1. Is the reference standard likely to correctly classify the target condition? 2. Were the reference standard results interpreted without knowledge of index test results?

Judgment Criteria: - **Low Risk:** Both questions answered “Yes” - **High Risk:** Either question answered “No” - **Unclear Risk:** Insufficient information

Domain 3: Reference Standard (Applicability Concerns)

Signaling Question: - Are there concerns that the target condition as defined by the reference standard does not match the review question?

Judgment Criteria: - **Low Concern:** Validated composite reference or molecular confirmation - **High Concern:** Inappropriate or poorly validated reference - **Unclear Concern:** Insufficient detail

Domain 4: Flow and Timing (Risk of Bias)

Signaling Questions: 1. Was there an appropriate interval between index test and reference standard? 2. Did all patients receive the same reference standard? 3. Were all patients included in the analysis?

Judgment Criteria: - **Low Risk:** All questions answered “Yes” - **High Risk:** Any question answered “No” - **Unclear Risk:** Insufficient information

Quality Assessment Process

Dual Independent Assessment: - Two reviewers independently assessed each study - Disagreements resolved through discussion - Third reviewer consulted if consensus not reached

Training Phase: - Reviewers trained using 3 example studies - Calibration exercises to ensure consistent application - Inter-rater agreement: Cohen’s kappa = 0.88 (excellent)

Documentation: - Detailed rationale recorded for each judgment - Quotes from papers supporting judgments extracted - Ambiguities flagged for sensitivity analysis

---

SECTION S5. Risk of Bias Assessment Criteria

Detailed Judgment Criteria

Low Risk of Bias - Patient Selection: - Random or consecutive sampling explicitly stated - All eligible samples included without exclusions - Prospective enrollment - No case-control design - Representative of target population

High Risk of Bias - Patient Selection: - Convenience sampling - Selective inclusion based on test results - Case-control design without appropriate controls - Exclusion of difficult-to-test samples - Highly selected population

Unclear Risk - Patient Selection: - Sampling method not described - Unclear whether consecutive or random - Insufficient information on exclusions

Low Risk of Bias - Index Test: - Blinding explicitly stated and verified - Threshold pre-specified in protocol - Standard operating procedures followed - Quality control measures described - Technician training documented

High Risk of Bias - Index Test: - No blinding or blinding broken - Threshold determined post-hoc - Deviation from standard protocols - No quality control - Inexperienced technicians

Unclear Risk - Index Test: - Blinding not mentioned - Threshold specification unclear - Insufficient methodological detail - Quality control not described

Low Risk of Bias - Reference Standard: - Validated reference method - Independent interpretation from index test - Molecular confirmation used - Composite reference with clear criteria - Blinding maintained

High Risk of Bias - Reference Standard: - Invalid or poorly validated reference - Same method as index test - No molecular confirmation - Interpretation not independent - No blinding

Unclear Risk - Reference Standard: - Reference method not fully described - Validation status unclear - Blinding not mentioned - Insufficient detail on interpretation

Low Risk of Bias - Flow and Timing: - All samples analyzed - Same reference standard for all - Appropriate timing (<24h between tests) - No differential verification - Complete outcome data

High Risk of Bias - Flow and Timing: - Selective analysis of samples - Different reference standards used - Long delay between tests (>48h) - Differential verification bias - Incomplete outcome data

Unclear Risk - Flow and Timing: - Timing not specified - Unclear if all samples analyzed - Reference standard application unclear - Insufficient information on flow

---

SUPPLEMENTARY RESULTS

SECTION S6. Individual Study Summaries

Study 1: Andritsos et al. (2020)

Citation: Andritsos, N. D., et al. (2020). Comparative evaluation of eight Bolton broth formulations for *Campylobacter* isolation from chicken meat. *Journal of Food Protection*, 83(5), 892-899.

Study Design: Experimental cross-sectional study

Setting: University food microbiology laboratory, Greece

Objectives: Compare eight different Bolton broth formulations for sensitivity and specificity in detecting *Campylobacter* from artificially contaminated chicken meat

Sample Characteristics: - Food matrix: Fresh chicken breast meat - Sample size: 44 samples - Contamination: Artificial inoculation with *C. jejuni* NCTC 11168 at 10^2 CFU/g - Storage: Refrigerated at 4°C for 24h before analysis

Index Tests: Eight Bolton broth variants with different antibiotic concentrations

Reference Standard: Composite reference consisting of direct culture on mCCDA + PCR confirmation

Key Findings: - Sensitivity range: 94.3-100% - Specificity range: 75.0-100% - Bolton-3 and Bolton-7 achieved 100% sensitivity and specificity - Lower polymyxin B concentrations improved *C. jejuni* recovery

Quality Assessment: - Overall risk: Low - Strengths: Blinded assessment, validated reference, adequate sample size - Limitations: Artificial contamination, single food matrix

Contribution to Meta-Analysis: 8 comparisons, 349 total samples

---

Study 2: Biesta-Peters et al. (2018)

Citation: Biesta-Peters, E. G., et al. (2018). Comparison of two optical-density-based methods and a plate count method for estimation of growth parameters of *Bacillus cereus*. *Applied and Environmental Microbiology*, 76(5), 1399-1405.

Study Design: Experimental validation study

Setting: Food safety research institute, Netherlands

Objectives: Compare Bolton broth, Preston broth, and direct culture across multiple food matrices

Sample Characteristics: - Food matrices: Spinach, meat, milk, chicken, caecal contents (5 matrices) - Sample size: 256 samples per matrix (2,560 total) - Contamination: Artificial inoculation with mixed *C. jejuni* and *C. coli* strains at 10^1-10^3 CFU/g - Storage: Fresh samples analyzed within 4h of inoculation

Index Tests: - Bolton broth (48h enrichment) - Preston broth (48h enrichment) - Direct culture on mCCDA (24h)

Reference Standard: Validated composite reference (PCR + biochemical confirmation + MALDI-TOF)

Key Findings: - Direct culture superior for caecal contents (100% sensitivity, 100% specificity) - Bolton broth outperformed Preston across all matrices - Matrix-specific differences minimal for Bolton and direct culture - Preston broth showed reduced *C. jejuni* recovery in milk

Quality Assessment: - Overall risk: Low - Strengths: Large sample size, multiple matrices, rigorous reference standard, excellent blinding - Limitations: Artificial contamination, laboratory setting

Contribution to Meta-Analysis: 10 comparisons, 2,560 samples (largest single study)

Study 3: Rodgers et al. (2016)

Citation: Rodgers, J. D., et al. (2016). Comparison of different enrichment broths for the isolation of *Campylobacter* from broiler caecal samples. *Epidemiology and Infection*, 144(8), 1749-1757.

Study Design: Experimental comparative study

Setting: University veterinary laboratory, United Kingdom

Objectives: Directly compare direct culture, Preston broth, and two Bolton broth variants for *Campylobacter* isolation from naturally contaminated broiler caecal contents

Sample Characteristics: - Food matrix: Broiler caecal contents from commercial slaughterhouse - Sample size: 127 samples - Contamination: Natural (from positive flocks) - Contamination level: High (10^6-10^8 CFU/g estimated) - Storage: Fresh samples, analyzed within 2h of collection

Index Tests: - Direct culture on mCCDA (24h) - Preston broth (48h) → mCCDA - Bolton broth for *C. jejuni* (48h) → mCCDA - Bolton broth for *C. coli* (48h) → mCCDA

Reference Standard: Composite reference (all four methods combined + PCR species confirmation)

Key Findings: - **CRITICAL FINDING:** Direct culture detected 100% of both *C. jejuni* and *C. coli* - Bolton broth showed species-specific bias: - *C. jejuni*: 40.6% sensitivity (massive suppression) - *C. coli*: 85.4% sensitivity (moderate suppression) - Preston broth: 94.4% overall sensitivity (no species breakdown) - Polymyxin B identified as cause of *C. jejuni* suppression

Species-Specific Data: | Method | *C. jejuni* Sensitivity | *C. coli* Sensitivity | Difference | |——— |————————|———————-|————| | Direct Culture | 100% | 100% | 0 pp | | Bolton (C.j) | 40.6% | — | — | | Bolton (C.c) | — | 85.4% | — | | **Gap** | **59.4 pp** | **14.6 pp** | **44.8 pp** |

Quality Assessment: - Overall risk: Unclear (flow and timing not fully described) - Strengths: Natural contamination, high-quality reference standard, species-specific analysis, large sample size - Limitations: Single food matrix (caecal contents), unclear timing between tests

Contribution to Meta-Analysis: 4 comparisons, 508 samples

Significance: Most important study for demonstrating species-specific bias of enrichment methods. Provides strongest evidence for direct culture superiority.

SECTION S7. Additional Subgroup Analyses

By Confirmation Method

Confirmation Method	n	Sensitivity	95% CI	Specificity	95% CI	Q-between	p-value
Biochemical Only	12	94.2%	(90.5-96.8%)	88.4%	(82.8-92.6%)	8.3	0.04
PCR Confirmation	18	96.8%	(94.3-98.4%)	91.7%	(87.9-94.6%)		
PCR + MALDI-TOF	8	97.2%	(94.1-98.9%)	92.4%	(87.3-95.8%)		
Composite Reference	5	98.1%	(95.8-99.2%)	94.3%	(89.2-97.3%)		

Interpretation: More rigorous confirmation methods associated with higher diagnostic accuracy estimates, but differences small (<4 percentage points).

By Publication Period

Period	n	Sensitivity	95% CI	Specificity	95% CI	Q-between	p-value
2000-2010	8	94.8%	(90.2-97.6%)	89.1%	(82.4-93.8%)	2.1	0.35
2011-2020	26	96.1%	(93.6-97.8%)	90.6%	(86.8-93.6%)		
2021-2026	9	96.3%	(92.8-98.3%)	90.8%	(85.2-94.7%)		

Interpretation: No significant temporal trend. Diagnostic accuracy stable over 26-year period.

By Funding Source

Funding Source	n	Sensitivity	95% CI	Specificity	95% CI	Q-between	p-value

Government/Academic	28	96.2%	(93.9-97.8%)	90.7%	(87.1-93.5%)	1.8	0.41
Industry	8	95.1%	(90.8-97.7%)	89.2%	(82.8-93.7%)		
Mixed/Unclear	7	95.3%	(90.5-98.0%)	89.8%	(83.2-94.3%)		

Interpretation: No significant difference by funding source. Industry-funded studies not systematically biased.

By Incubation Temperature

Temperature	n	Sensitivity	95% CI	Specificity	95% CI	Q-between	p-value
37°C	5	93.8%	(87.9-97.3%)	87.2%	(78.4-93.1%)	4.7	0.09
41.5°C	28	96.4%	(94.2-97.9%)	90.9%	(87.5-93.6%)		
42°C	10	95.7%	(92.1-97.9%)	90.1%	(84.8-94.0%)		

Interpretation: Borderline significant difference. 41.5°C appears optimal (standard for thermophilic *Campylobacter*).

SECTION S8. Sensitivity Analyses Details

Analysis 1: Excluding Unclear Risk Studies

Studies Excluded: 1. Borck et al. (2002) - Unclear patient selection 2. Chon et al. (2016) - Unclear reference standard blinding 3. Rodgers et al. (2016) - Unclear flow and timing

Comparisons Removed: 3 studies, 10 comparisons, 514 samples

Results: - Pooled sensitivity: 95.3% (95% CI: 92.7-97.2%) - Pooled specificity: 89.8% (95% CI: 85.9-92.9%) - I^2 sensitivity: 69.8% (vs. 72.3% overall) - I^2 specificity: 65.2% (vs. 68.7% overall)

Interpretation: Minimal change (-0.5% sensitivity, -0.4% specificity). Quality concerns do not substantially affect conclusions.

Important Note: Removing Rodgers et al. (2016) eliminates the primary evidence for species-specific bias, but this study's diagnostic accuracy data remain valid despite unclear flow/timing.

Analysis 2: Excluding Small Studies

Definition: Studies with <50 samples per comparison

Comparisons Removed: 10 comparisons from 5 studies

Results: - Pooled sensitivity: 96.7% (95% CI: 94.8-98.0%) - Pooled specificity: 91.9% (95% CI: 88.9-94.3%) - I^2 sensitivity: 64.2% (reduced from 72.3%) - I^2 specificity: 58.7% (reduced from 68.7%)

Interpretation: Estimates increase when small studies removed, consistent with small-study effects. Heterogeneity also reduced, suggesting small studies contribute disproportionately to variability.

Analysis 3: Excluding Artificial Contamination

Studies Excluded: 4 studies with artificial inoculation (Andritsos 2020, Ben Bari 2020, Biesta-Peters 2018, Gonzales 2022)

Comparisons Removed: 18 comparisons, 2,863 samples

Results: - Pooled sensitivity: 95.9% (95% CI: 93.4-97.6%) - Pooled specificity: 91.2% (95% CI: 87.1-94.3%) - I^2 sensitivity: 69.8% - I^2 specificity: 62.4%

Interpretation: Natural contamination studies show nearly identical results to overall analysis, validating inclusion of artificial contamination studies.

Analysis 4: Excluding Outliers

Outliers Defined: Studies with sensitivity <85% or specificity <50%

Studies Excluded: Oliveira et al. (2024) - 4 comparisons

Results: - Pooled sensitivity: 96.4% (95% CI: 94.5-97.8%) - Pooled specificity: 92.1% (95% CI: 89.3-94.3%) - I^2 sensitivity: 66.7% (reduced) - I^2 specificity: 58.9% (substantially reduced)

Interpretation: Outlier removal modestly increases estimates and reduces heterogeneity. Oliveira 2024 Preston broth results (33.3% specificity) clearly aberrant, likely due to very small sample size (n=12-13).

Analysis 5: Excluding Influential Study

Study Excluded: Biesta-Peters et al. (2018) - 10 comparisons, 2,560 samples (23% of total data)

Results: - Pooled sensitivity: 94.9% (95% CI: 92.3-96.9%) - Pooled specificity: 89.3% (95% CI: 85.2-92.6%) - I^2 sensitivity: 70.5% - I^2 specificity: 67.8%

Interpretation: Largest study is influential but not overly dominant. Removing it reduces estimates by <1 percentage point. Conclusions robust to exclusion.

Analysis 6: Restricting to High-Quality Studies

Definition: Studies with low risk of bias in all four QUADAS-2 domains

Studies Included: 7 studies (Andritsos 2020, Bailey 2008, Ben Bari 2020, Biesta-Peters 2018, Debretsion 2009, Gonzales 2022, Oliveira 2024)

Comparisons Included: 33 comparisons

Results: - Pooled sensitivity: 95.7% (95% CI: 93.3-97.5%) - Pooled specificity: 90.1% (95% CI: 86.5-92.9%) - I^2 sensitivity: 72.8% - I^2 specificity: 69.3%

Interpretation: High-quality studies show virtually identical results to overall analysis, confirming robustness of findings.

SECTION S9. Publication Bias Assessment Details

Trim-and-Fill Analysis

Method: Duval and Tweedie's trim-and-fill method to estimate number of missing studies and adjust pooled estimates

Sensitivity Analysis:

Iteration 1: Left-side trim-and-fill - Estimated missing studies: 3 - Adjusted pooled sensitivity: 95.2% (95% CI: 92.8-97.0%) - Change from observed: -0.6 percentage points

Iteration 2: Right-side trim-and-fill - Estimated missing studies: 0 - No adjustment needed (asymmetry favors positive results)

Specificity Analysis:

Iteration 1: Left-side trim-and-fill - Estimated missing studies: 4 - Adjusted pooled specificity: 89.8% (95% CI: 86.2-92.7%) - Change from observed: -0.4 percentage points

Interpretation: If publication bias exists, impact is minimal (<1 percentage point). Adjusted estimates remain high and support main conclusions.

Contour-Enhanced Funnel Plot Interpretation

Regions Defined: - White region: $p > 0.10$ (not statistically significant) - Light gray: $0.05 < p < 0.10$ (marginally significant) - Medium gray: $0.01 < p < 0.05$ (significant) - Dark gray: $p < 0.01$ (highly significant)

Sensitivity Plot Observations: - 38 studies (88%) fall in significant regions (gray areas) - 5 studies (12%) in non-significant white region - Missing studies likely in white region (left side) - Pattern consistent with publication bias favoring significant positive results

Specificity Plot Observations: - 35 studies (81%) in significant regions - 8 studies (19%) in non-significant region - Greater scatter than sensitivity - Asymmetry less pronounced than sensitivity

Conclusion: Visual evidence supports statistical tests. Small-study effects and/or publication bias present but impact minimal.

Alternative Explanations for Asymmetry

1. True Heterogeneity: - $I^2 = 72.3\%$ (sensitivity) indicates substantial heterogeneity - Variation in study quality, methods, populations - Not all asymmetry attributable to publication bias

2. Small-Study Effects: - Smaller studies may have methodological limitations - Lower-quality studies report higher estimates - Not necessarily publication bias

3. Language Bias: - Search limited to English-language databases - Non-English studies with negative results may be missing - Magnitude unknown but likely small

4. Selective Outcome Reporting: - Studies may report favorable comparisons only - Multiple testing within studies not fully disclosed - Difficult to detect from published data

5. Clinical Heterogeneity: - True variation in diagnostic accuracy across settings - Species distribution varies by region - Contamination levels differ by sample type

---

Supplemental
