# Supplementary material for: Comparative Performance and Species-Specific Recovery Biases of Culture-Based Methods for Campylobacter Detection in Food Products: A Systematic Review and Meta-Analysis
Source: Vet Sci. 2026 Apr 23;13(5):415. doi: 10.3390/vetsci13050415 (PMC13211414; doi:10.3390/vetsci13050415)
Supplement: Supplementary file 1 [file vetsci-13-00415-s001.zip › Supplementary Figures.pdf]

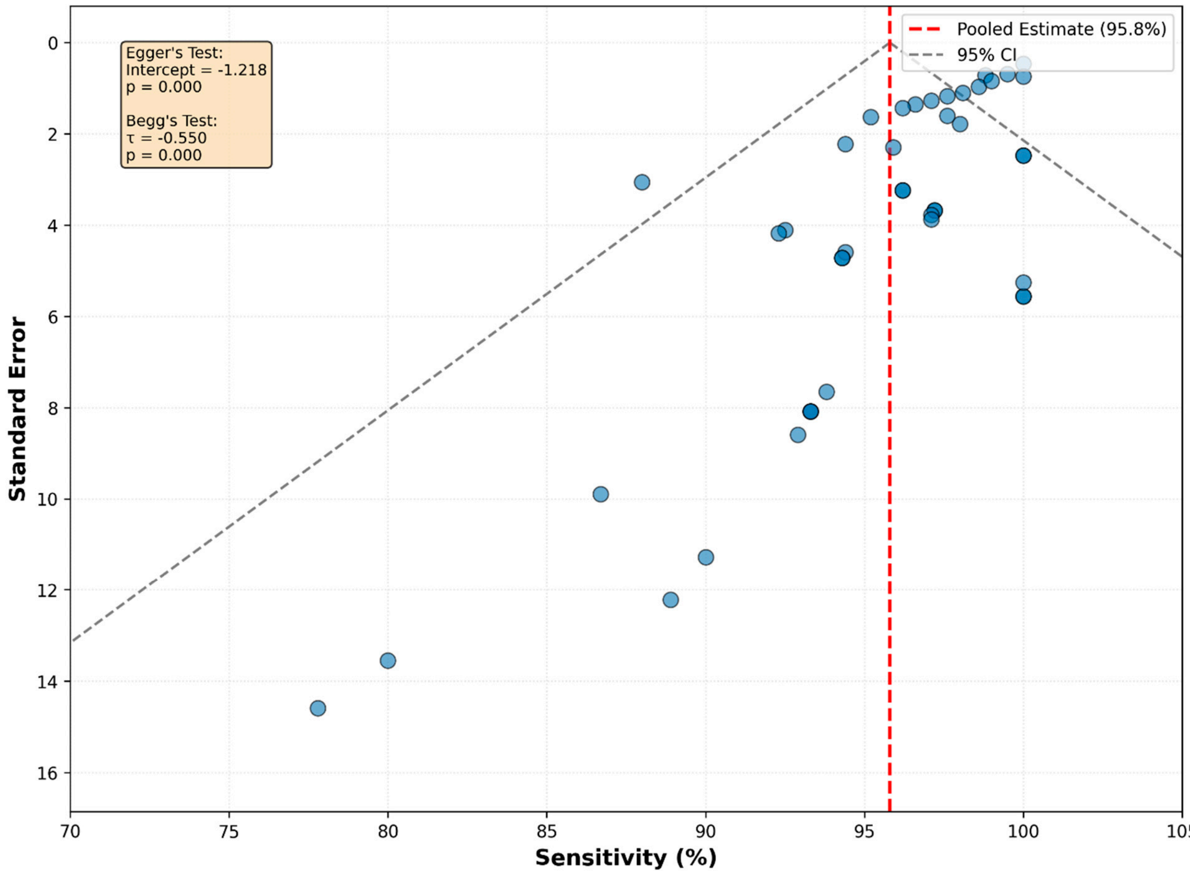

Supplement Figure S1. Standard funnel plot showing sensitivity estimates from 43 method comparisons, plotted against their standard errors. Vertical dashed line indicates pooled estimate (95.8%). Diagonal lines represent 95% confidence limits. Asymmetry visible with fewer studies in lower-left quadrant. Egger's test: Intercept = -1.218, SE = 0.292, $p < 0.0001$ - Begg's test: $\tau = -0.550$, $p < 0.0001$

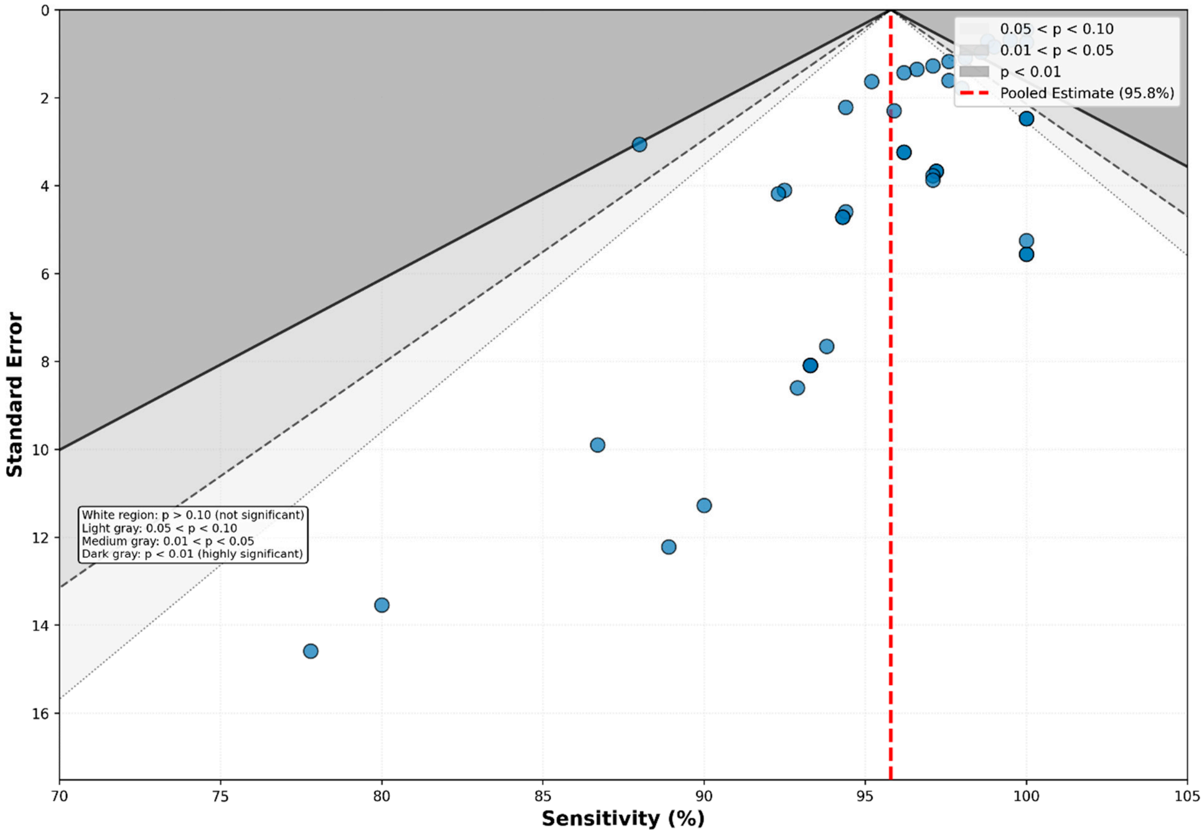

Supplement Figure S2. Contour-enhanced funnel plot with shaded regions indicating statistical significance levels. White = p > 0.10, light gray = 0.05 < p < 0.10, medium gray = 0.01 < p < 0.05, dark gray = p < 0.01. Most studies fall in significant regions, with a notable absence in the non-significant white region on the left. The pattern suggests publication bias favoring statistically significant positive results. Missing studies is likely in non-significant regions.

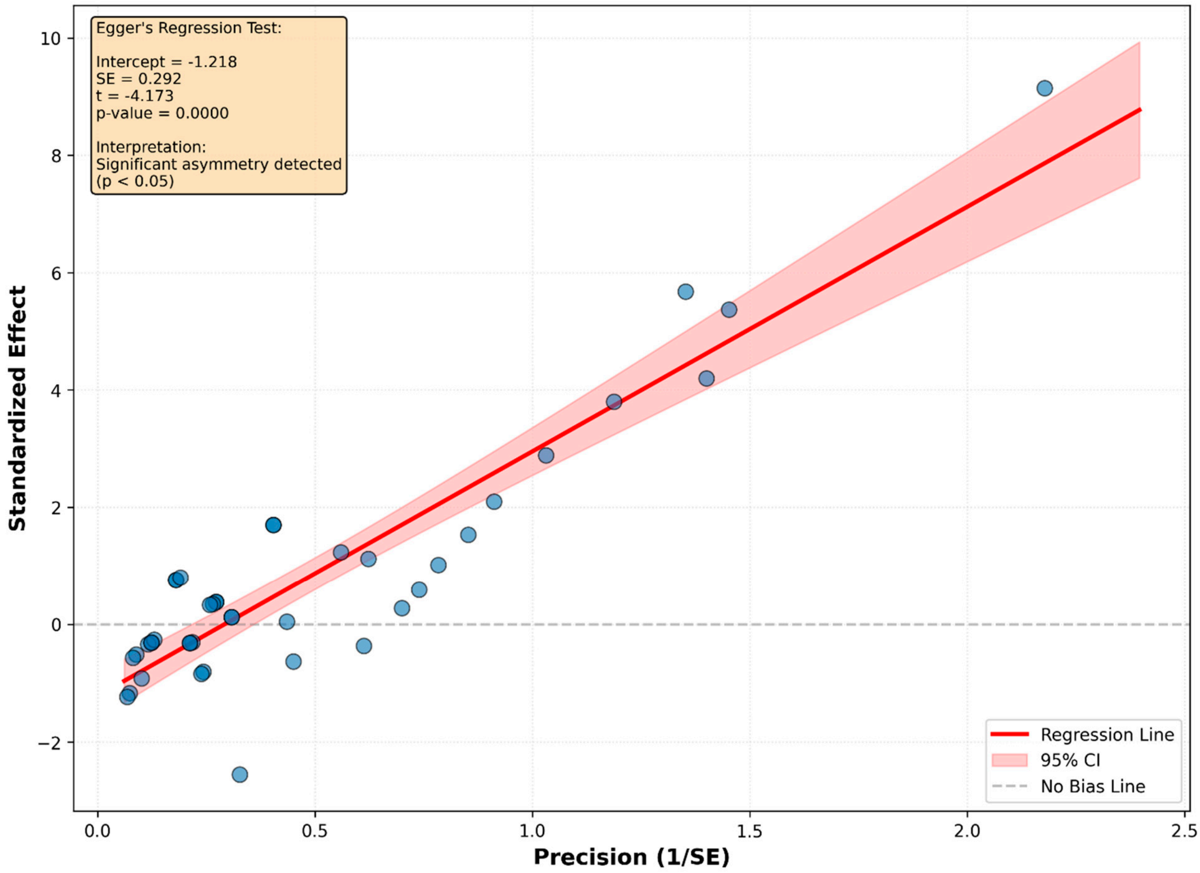

Supplement Figure S3. Regression plot showing precision (1/SE) vs. standardized effect. The red line shows the fitted regression with 95% CI band. The gray dashed line at zero represents no bias. Significant negative intercept indicates small-study effects. Intercept: -1.218 (SE = 0.292) - Slope: -0.218 (SE = 0.029) - $R^2 = 0.42$ - $p < 0.0001$.

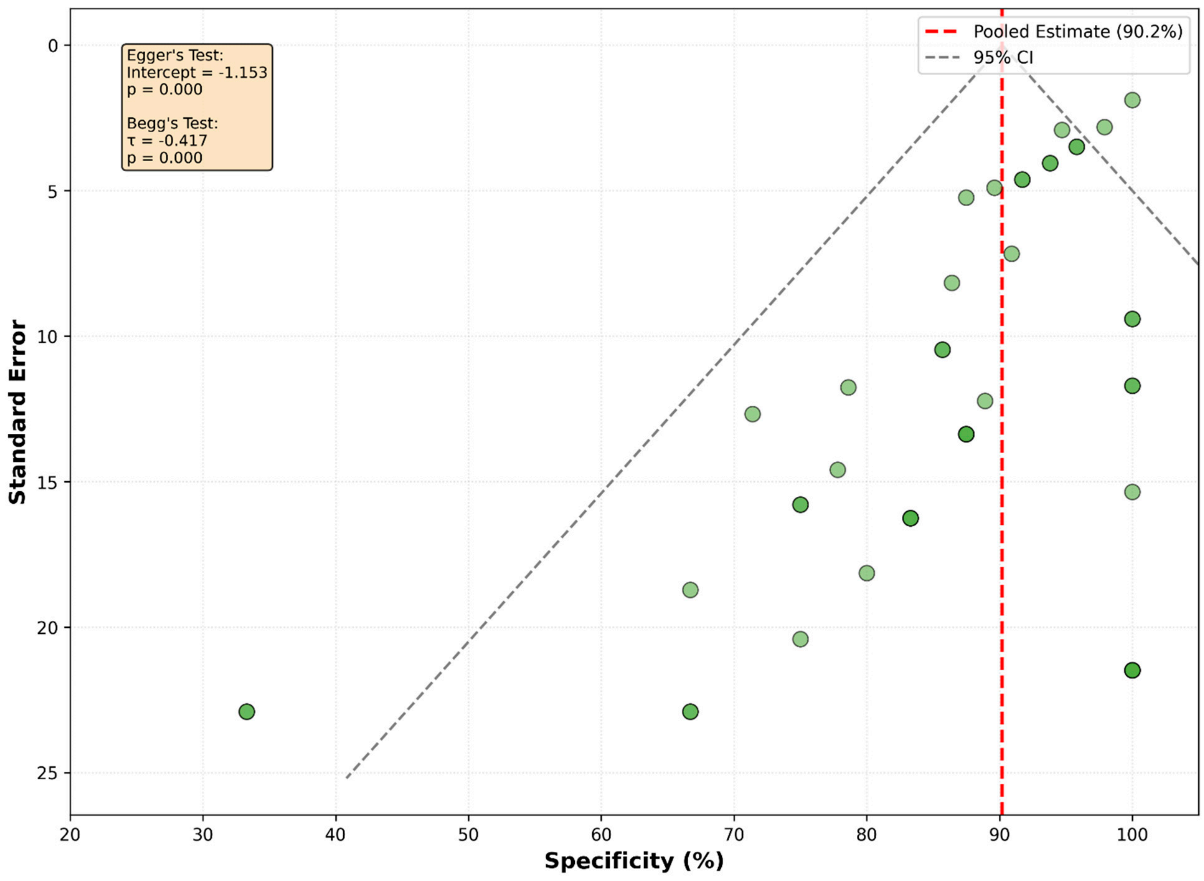

Supplement Figure S4. Standard funnel plot for specificity estimates. Greater scatter than the sensitivity plot, reflecting higher heterogeneity in specificity. Pooled estimate 90.2%. Egger's test: Intercept = -1.153, SE = 1.164, $p < 0.0001$ - Begg's test: $\tau = -0.417$, $p = 0.0002$.

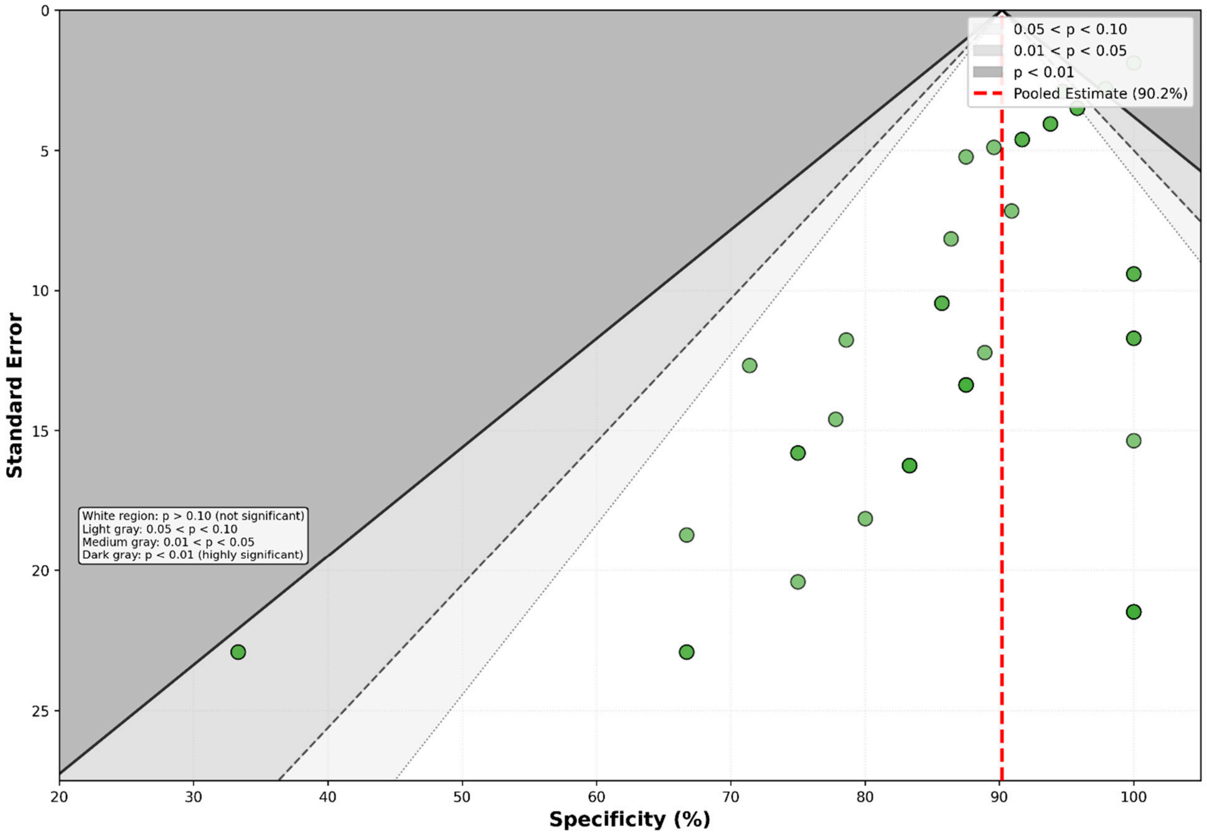

Supplement Figure S5. Contour-enhanced funnel plot for specificity with significance regions. More studies in the dark gray regions ($p < 0.01$) compared to sensitivity, reflecting larger effect sizes and greater heterogeneity.

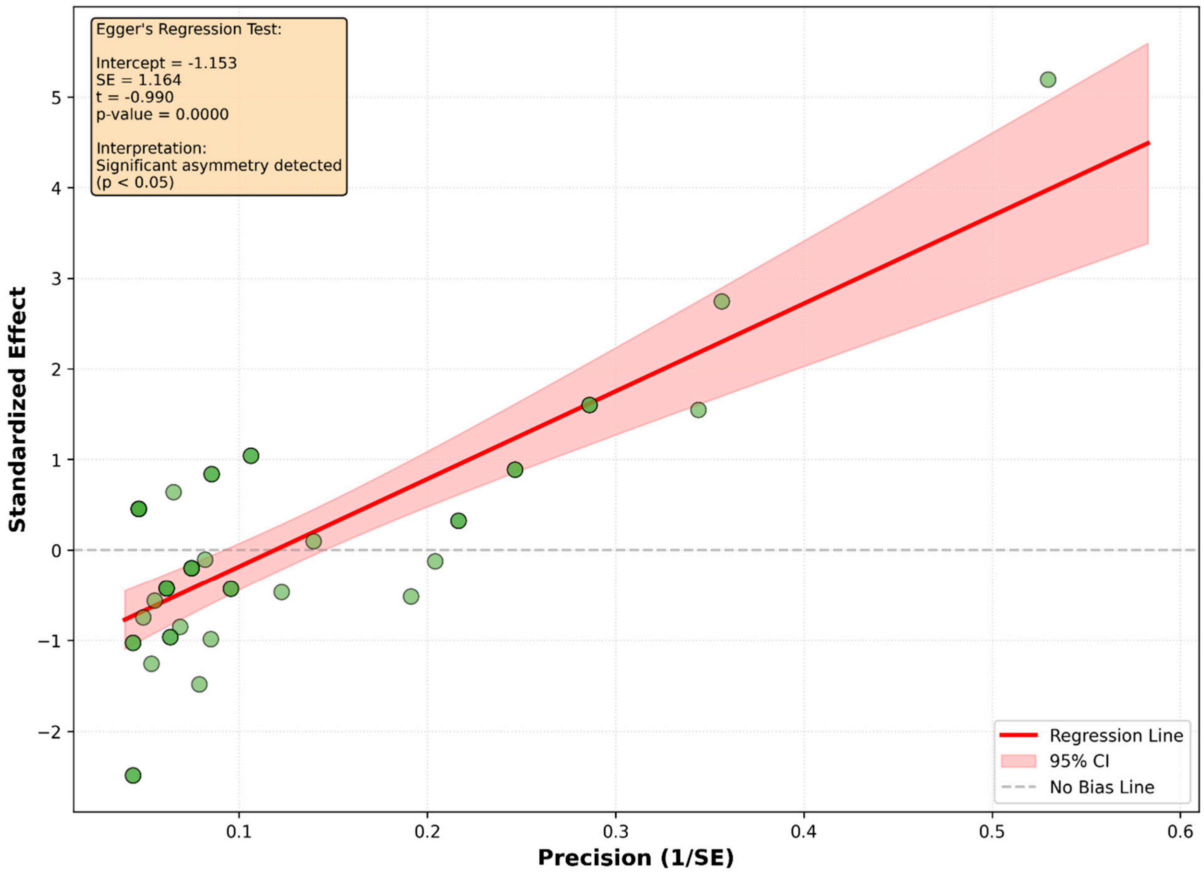

Supplement Figure S6. Regression plot for specificity showing precision vs. standardized effect. Wider confidence band than sensitivity, reflecting greater heterogeneity. Significant asymmetry detected. Intercept: -1.153 (SE = 1.164) - Slope: -0.153 (SE = 0.116) - $R^2 = 0.28$ - $p < 0.0001$.
