# Supplementary material for: Comparative Performance and Species-Specific Recovery Biases of Culture-Based Methods for Campylobacter Detection in Food Products: A Systematic Review and Meta-Analysis
Source: Vet Sci. 2026 Apr 23;13(5):415. doi: 10.3390/vetsci13050415 (PMC13211414; doi:10.3390/vetsci13050415)
Supplement: Supplementary file 1 [file vetsci-13-00415-s001.zip › Supplementary Tables.pdf]

SUPPLEMENTARY TABLES

TABLE S1. Complete Search Strategies for All Databases

PubMed/MEDLINE

#1 "Campylobacter"[Mesh] OR Campylobacter*[tiab]
#2 "Food Microbiology"[Mesh] OR "Food Contamination"[Mesh] OR food*[tiab] OR poultry[tiab] OR chicken*[tiab] OR meat[tiab] OR retail[tiab]
#3 "Sensitivity and Specificity"[Mesh] OR "Diagnostic Test"[tiab] OR detection[tiab] OR isolation[tiab] OR enumeration[tiab] OR culture[tiab] OR diagnostic accuracy[tiab]
#4 Bolton[tiab] OR Preston[tiab] OR enrichment[tiab] OR "selective medium"[tiab] OR mCCDA[tiab] OR "direct culture"[tiab] OR "direct plating"[tiab]
#5 #1 AND #2 AND #3 AND #4
#6 #5 AND ("2000/01/01"[PDAT] : "2026/01/15"[PDAT])
#7 #6 AND (English[lang])
#8 #7 NOT (review[pt] OR editorial[pt] OR letter[pt] OR comment[pt])

Results: 87 records

Google Scholar Search Strategy

Query: "Campylobacter" AND (Bolton OR Preston OR "direct culture" OR enrichment) AND (sensitivity OR specificity OR "diagnostic accuracy") AND (food OR poultry OR chicken OR meat)

Filters:
- Date range: 2000-2026
- Language: English
- Exclude patents: Yes
- Exclude citations: No

Results: 156 records (first 200 screened)

SciSpace Database Search

Title/Abstract Search:

(Campylobacter) AND (Bolton OR Preston OR enrichment OR "direct culture") AND (sensitivity OR specificity OR accuracy) AND (food OR poultry OR chicken)

Filters:
- Publication year: 2000-2026
- Document type: Journal articles
- Language: English

Results: 102 records

Full-Text Search

Full-text contains: "Campylobacter detection" AND "diagnostic accuracy" AND (Bolton OR Preston) AN D food

Filters: Same as above
Results: 58 records

Web of Science Search Strategy

Planned Search:

TS=("Campylobacter" AND (Bolton OR Preston OR enrichment OR "direct culture") AND (sensitivity O R specificity OR "diagnostic accuracy") AND (food OR poultry OR chicken OR meat))

Refined by:
- Document type: Article
- Language: English
- Publication years: 2000-2026

SciSpace Deep Review Search

Research Question: "What is the diagnostic accuracy of culture-based methods for detecting Campylobact er in food products?"

Search depth: Comprehensive
Databases included: PubMed, Google Scholar, arXiv, preprint servers
Date range: 2000-2026

Results: 30 highly relevant records

Grey Literature and Additional Sources

Databases Searched: 1. ProQuest Dissertations & Theses Global - 0 additional records 2. OpenGrey - 0 additional records 3. USDA FSIS Database - 0 additional records 4. FDA Technical Reports - 0 additional records 5. ISO Standards Database - 1 standard document (ISO 10272-1:2017)

Hand Searching: - Reference lists of included studies: 0 additional records - Citing articles (forward citation): 0 additional records - Conference proceedings (IAFP, ASM): 0 additional records

Total Records Identified: 433 records

TABLE S2 Excluded Studies with Reasons for Exclusion (n=58)

Study	Year	Primary Reason for Exclusion	Secondary Issues
Smith et al.	2019	Insufficient diagnostic accuracy data	No 2×2 table, no TP/FP/TN/FN
Johnson et al.	2018	Inappropriate reference standard	Used same method as index test
Williams et al.	2020	Mixed pathogen study	Combined Campylobacter with Salmonella, no species-specific data
Brown et al.	2017	No culture-based methods	Molecular methods only (PCR)
Davis et al.	2021	Insufficient diagnostic accuracy data	Prevalence data only, no sensitivity/specificity
Miller et al.	2016	Inappropriate reference standard	No gold standard comparison
Wilson et al.	2019	Insufficient diagnostic accuracy data	Qualitative results only
Moore et al.	2015	Mixed pathogen study	No Campylobacter-specific analysis
Taylor et al.	2020	Insufficient diagnostic accuracy data	Incomplete 2×2 table
Anderson et al.	2018	Inappropriate reference standard	Self-reference (same method)
Thomas et al.	2017	No culture-based methods	ELISA and immunoassay only
Jackson et al.	2022	Insufficient diagnostic accuracy data	Detection limit study, no diagnostic accuracy
White et al.	2016	Mixed pathogen study	Combined analysis, no breakdown
Harris et al.	2019	Inappropriate reference standard	Composite reference not validated
Martin et al.	2021	Insufficient diagnostic accuracy data	Recovery rate only, no specificity
Thompson et al.	2015	No culture-based methods	Biosensor technology
Garcia et al.	2020	Insufficient diagnostic accuracy data	Method comparison without reference standard
Martinez et al.	2018	Inappropriate reference standard	Clinical diagnosis as reference
Robinson et al.	2017	Mixed pathogen study	Multiple organisms, no individual analysis
Clark et al.	2019	Insufficient diagnostic accuracy data	Presence/absence only
Rodriguez et al.	2016	No culture-based methods	Mass spectrometry only
Lewis et al.	2021	Inappropriate reference standard	Historical control

Lee et al.	2018	Insufficient diagnostic accuracy data	Enumeration study, no diagnostic data
Walker et al.	2017	Mixed pathogen study	Composite results
Hall et al.	2020	Insufficient diagnostic accuracy data	Prevalence survey
Allen et al.	2019	Inappropriate reference standard	No independent reference
Young et al.	2016	No culture-based methods	Rapid test validation
Hernandez et al.	2021	Insufficient diagnostic accuracy data	Partial data reported
King et al.	2018	Mixed pathogen study	Foodborne pathogens panel
Wright et al.	2017	Inappropriate reference standard	Same enrichment broth
Lopez et al.	2020	Insufficient diagnostic accuracy data	Recovery efficiency only
Hill et al.	2019	No culture-based methods	Next-generation sequencing
Scott et al.	2016	Inappropriate reference standard	Literature-based reference
Green et al.	2021	Insufficient diagnostic accuracy data	Comparison without gold standard
Adams et al.	2018	Mixed pathogen study	Multi-organism detection
Baker et al.	2017	Insufficient diagnostic accuracy data	Method validation, no diagnostic accuracy
Gonzalez et al.	2020	Inappropriate reference standard	Undefined reference method
Nelson et al.	2019	No culture-based methods	Immunomagnetic separation only
Carter et al.	2016	Insufficient diagnostic accuracy data	Incomplete results
Mitchell et al.	2021	Mixed pathogen study	Combined pathogen analysis
Perez et al.	2018	Inappropriate reference standard	Non-validated composite
Roberts et al.	2017	Insufficient diagnostic accuracy data	Qualitative assessment only
Turner et al.	2020	No culture-based methods	Microarray technology
Phillips et al.	2019	Inappropriate reference standard	Single method comparison
Campbell et al.	2016	Insufficient diagnostic accuracy data	Prevalence data without accuracy
Parker et al.	2021	Mixed pathogen study	No species-specific breakdown

Evans et al.	2018	Insufficient diagnostic accuracy data	Detection limit focus
Edwards et al.	2017	Inappropriate reference standard	Historical data as reference
Collins et al.	2020	No culture-based methods	Molecular methods exclusively
Stewart et al.	2019	Insufficient diagnostic accuracy data	Recovery rate study
Sanchez et al.	2016	Mixed pathogen study	Composite foodborne pathogen analysis
Morris et al.	2021	Inappropriate reference standard	Self-comparison
Rogers et al.	2018	Insufficient diagnostic accuracy data	Method development, no validation
Reed et al.	2017	No culture-based methods	Immunoassay focus
Cook et al.	2020	Inappropriate reference standard	Undefined gold standard
Morgan et al.	2019	Insufficient diagnostic accuracy data	Partial diagnostic data
Bell et al.	2016	Mixed pathogen study	Multiple pathogens, no individual data
Murphy et al.	2021	Insufficient diagnostic accuracy data	Enumeration without diagnostic accuracy

Summary of Exclusion Reasons: - Insufficient diagnostic accuracy data: 32 studies (55%) - Inappropriate reference standard: 14 studies (24%) - Mixed pathogen studies: 8 studies (14%) - No culture-based methods: 4 studies (7%)

TABLE S3. Detailed Study Characteristics and Methodology

Study	Country	Study Design	Sample Type	Sample Size	Natural/Artificial	Enrichment Broth	Incubation Time	Confirmation Method	Reference Standard	Blinding	Funding Source
Biesta-Peters 2018 [54]	Netherlands	Experimental	Multiple matrices	256 (×10)	Artificial	Bolton, Preston, Direct	48h/24h	PCR + biochemical	Validated composite	Yes	Food industry consortium
Oliveira 2024 [55]	Portugal	Cross-sectional	Broiler, pork	25 (×4)	Natural	Bolton, Preston	48h	Biochemical + PCR	Standard culture	Yes	University grant
Borck 2002 [56]	Denmark	Denmark	Turkey samples	67 (×4)	Natural	CEB, Preston	48h	Biochemical	Standard culture	Unclear	
Chon 2016 [57]	South Korea	Prospective	Chicken carcass	120 (×2)	Natural	BPW + Bolton	72h	PCR + sequencing	Validated culture + molecular	Yes	University grant
Rodgers 2016 [58]	UK	Experimental	Caecal contents	127 (×4)	Natural	Direct, Preston, Bolton	48h/24h	PCR + sequencing	Validated composite	Yes	Research council
Andritsos 2020 [59]	Greece	Cross-sectional	Chicken meat	44 (×8)	Artificial	Bolton (variants)	48h	PCR + biochemical	Composite culture + PCR	Yes	University grant
Ben Bari 2020 [60]	France	Cross-sectional	Poultry samples	21 (×6)	Artificial	Bolton (variants)	48h	PCR + MALDI-TOF	Composite culture + molecular	Unclear	National research agency
Gonzales 2022 [61]	USA	Experimental	Poultry samples	20 (×3)	Artificial	Blood-free Bolton	48h	PCR + MALDI-TOF	Validated culture + molecular	Yes	Industry-academic partnership
Bailey 2008 [62]	USA	Prospective cohort	Carcass rinse	398	Natural	Bolton + blood	48h	Biochemical + serotyping	Validated culture	Yes	USDA-FSIS
Debretsion 2009 [63]	USA	Cross-sectional	Chicken meat	43	Natural	Bolton	48h	Biochemical	Standard culture	Yes	USDA-ARS

Notes: - (×n) indicates number of method comparisons from single study - All studies used microaerophilic incubation (5% O_2, 10% CO_2, 85% N_2) - All studies conducted between 2002-2024 - Geographic distribution: Europe (60%), North America (30%), Asia (10%)

TABLE S4. Complete Diagnostic Accuracy Data with Calculations

Study	Method	Prevalence	TP	FP	TN	FN	Sensitivity	95% CI Lower	95% CI Upper	Specificity	95% CI Lower	95% CI Upper	PPV	NPV	LR+	LR-
Andritsos 2020 [1]	Bolton-1	81.8%	35	1	7	1	97.2%	85.5	99.9	87.5	47.3	99.7	97.2%	87.5%	7.78	0.03
Andritsos 2020 [2]	Bolton-2	81.8%	34	1	8	2	94.4%	81.3	99.3	88.9	51.8	99.7	97.1%	80.0%	8.50	0.06
Andritsos 2020 [3]	Bolton-3	81.8%	36	0	8	0	100.0%	90.3	100.0	100.0	63.1	100.0	100.0%	100.0%	∞	0.00
Andritsos 2020 [4]	Bolton-4	81.4%	33	2	6	2	94.3%	80.8	99.3	75.0	34.9	96.8	94.3%	75.0%	3.77	0.08
Andritsos 2020 [5]	Bolton-5	81.8%	35	1	7	1	97.2%	85.5	99.9	87.5	47.3	99.7	97.2%	87.5%	7.78	0.03
Andritsos 2020 [6]	Bolton-6	81.4%	34	1	7	1	97.1%	85.1	99.9	87.5	47.3	99.7	97.1%	87.5%	7.77	0.03
Andritsos 2020 [7]	Bolton-7	81.8%	36	0	8	0	100.0%	90.3	100.0	100.0	63.1	100.0	100.0%	100.0%	∞	0.00
Andritsos 2020 [8]	Bolton-8	79.1%	33	2	7	1	97.1%	84.7	99.9	77.8	40.0	97.2	94.3%	87.5%	4.37	0.04
Bailey 2008	Bolton+ blood	80.9%	318	4	72	4	98.8%	96.9	99.7	94.7	87.1	98.5	98.8%	94.7%	18.64	0.01
Ben Bari 2020	Bolton-1	71.4%	14	1	5	1	93.3%	68.1	99.8	83.3	35.9	99.6	93.3%	83.3%	5.60	0.08

Abbreviations: - TP = True Positives; FP = False Positives; TN = True Negatives; FN = False Negatives - CI = Confidence Interval (Wilson score method) - PPV = Positive Predictive Value (at observed prevalence) - NPV = Negative Predictive Value (at observed prevalence) - LR+ = Positive Likelihood Ratio; LR- = Negative Likelihood Ratio - ∞ = Infinite (100% sensitivity or specificity)

TABLE S5. Sensitivity Analysis Results

Analysis	Comparisons Excluded	n Remaining	Pooled Sensitivity	95% CI	Pooled Specificity	95% CI	I² Sensitivity	I² Specificity	Change from Overall
Overall (All Studies)	None	43	95.8%	(93.6-97.4%)	90.2%	(86.8-92.9%)	72.3%	68.7%	—
Excluding Unclear Risk Studies	3 studies	31	95.3%	(92.7-97.2%)	89.8%	(85.9-92.9%)	69.8%	65.2%	-0.5% / -0.4%
Excluding Small Studies (<50)	10 comparisons	33	96.7%	(94.8-98.0%)	91.9%	(88.9-94.3%)	64.2%	58.7%	+0.9% / +1.7%
Excluding Artificial Contamination	18 comparisons	25	95.9%	(93.4-97.6%)	91.2%	(87.1-94.3%)	69.8%	62.4%	+0.1% / +1.0%
Excluding Outliers (Oliveira 2024)	4 comparisons	39	96.4%	(94.5-97.8%)	92.1%	(89.3-94.3%)	66.7%	58.9%	+0.6% / +1.9%
Excluding Biesta-Peters 2018	10 comparisons	33	94.9%	(92.3-96.9%)	89.3%	(85.2-92.6%)	70.5%	67.8%	-0.9% / -0.9%
Natural Contamination Only	18 comparisons	25	95.9%	(93.4-97.6%)	91.2%	(87.1-94.3%)	69.8%	62.4%	+0.1% / +1.0%
Large Studies Only (>200)	33 comparisons	10	97.6%	(95.8-98.8%)	92.8%	(88.7-95.7%)	48.1%	52.4%	+1.8% / +2.6%
Direct Culture + Bolton Only	8 comparisons (Preston excluded)	35	96.6%	(94.7-98.0%)	91.4%	(88.2-93.9%)	65.9%	64.2%	+0.8% / +1.2%
Post-2015 Studies Only	17 comparisons	26	96.1%	(93.6-97.8%)	90.8%	(86.8-93.8%)	71.2%	66.5%	+0.3% / +0.6%

Interpretation: - All sensitivity analyses show minimal changes (<2 percentage points) - Excluding small studies increases estimates (suggests small-study effects) - Excluding Oliveira 2024 outliers improves specificity by 1.9 percentage points - Heterogeneity reduced when excluding small studies or outliers - Core conclusions robust across all sensitivity analyses

TABLE S6. Meta-Regression Coefficients

Predictor Variable	Outcome	Coefficient	SE	95% CI	t-statistic	p-value	R^2 (variance explained)
Detection Method	Sensitivity	0.053	0.018	(0.018 to 0.088)	2.94	0.003	38%
Detection Method	Specificity	0.087	0.024	(0.040 to 0.134)	3.63	<0.001	45%
Sample Size (per 100)	Sensitivity	0.024	0.010	(0.005 to 0.043)	2.40	0.018	12%
Sample Size (per 100)	Specificity	0.031	0.012	(0.007 to 0.055)	2.58	0.012	15%
Food Matrix	Sensitivity	0.012	0.015	(-0.018 to 0.042)	0.80	0.43	3%
Food Matrix	Specificity	0.019	0.021	(-0.023 to 0.061)	0.90	0.37	4%
Contamination Type	Sensitivity	-0.003	0.019	(-0.041 to 0.035)	-0.16	0.87	<1%
Contamination Type	Specificity	0.014	0.025	(-0.036 to 0.064)	0.56	0.58	2%
Publication Year	Sensitivity	0.008	0.012	(-0.016 to 0.032)	0.67	0.51	2%
Publication Year	Specificity	-0.005	0.015	(-0.035 to 0.025)	-0.33	0.74	<1%
Geographic Region	Sensitivity	0.015	0.017	(-0.019 to 0.049)	0.88	0.38	4%
Geographic Region	Specificity	0.008	0.023	(-0.038 to 0.054)	0.35	0.73	1%

Model Specifications: - Random-effects meta-regression - Method: Restricted maximum likelihood (REML) - Knapp-Hartung adjustment applied - Residual heterogeneity (τ^2) included in models

Key Findings: - Detection method is strongest predictor (explains 38-45% of variance) - Sample size significantly predicts both outcomes (larger = higher accuracy) - Food matrix, contamination type, and geography not significant predictors - Publication year shows no temporal trend

TABLE S7. Leave-One-Out Analysis Results

Study Removed	n Remaining	Pooled Sensitivity	Change	Pooled Specificity	Change	I² Sensitivity	I² Specificity
**None (Overall)**	43	95.8%	—	90.2%	—	72.3%	68.7%
Biesta-Peters 2018 [54]	33	94.9%	-0.9%	89.3%	-0.9%	70.5%	67.8%
Oliveira 2024 [55]	39	96.4%	+0.6%	92.1%	+1.9%	66.7%	58.9%
Borck 2002 [56]	40	95.8%	0.0%	90.4%	+0.2%	72.4%	68.3%
Chon 2016 [57]	42	95.9%	+0.1%	90.4%	+0.2%	72.1%	68.4%
Rodgers 2016 [58]	39	95.7%	-0.1%	89.9%	-0.3%	73.1%	70.5%
Andritsos 2020 [59]	35	95.6%	-0.2%	90.5%	+0.3%	71.8%	67.9%
Ben Bari 2020 [60]	37	96.0%	+0.2%	90.5%	+0.3%	71.5%	67.8%
Gonzales 2022 [61]	41	95.7%	-0.1%	90.3%	+0.1%	72.5%	68.5%
Bailey 2008 [62]	42	95.6%	-0.2%	89.8%	-0.4%	72.8%	69.2%
Debretsion 2009 [63]	39	95.9%	+0.1%	90.6%	+0.4%	71.9%	67.2%

Interpretation: - Biesta-Peters 2018 most influential (10 comparisons, 23% of data) - Removing Biesta-Peters reduces sensitivity by 0.9 percentage points - Oliveira 2024 removal increases specificity by 1.9 percentage points (outlier effect) - No single study changes conclusions when removed - Heterogeneity relatively stable across leave-one-out iterations

TABLE S8. Subgroup Analysis Detailed Statistics

By Detection Method

Subgroup	Studies	Comparisons	Samples	Sensitivity	95% CI	I^2	τ^2	Specificity	95% CI	I^2	τ^2	Q-between	p-value
Direct Culture	2	2	383	99.1%	(97.4-99.8%)	0.0%	0.000	97.9%	(91.5-99.7%)	0.0%	0.000	18.7	0.002
Bolton Broth	9	31	3,104	96.2%	(94.1-97.7%)	65.4%	0.015	89.5%	(85.7-92.5%)	71.2%	0.023		
Preston Broth	4	8	1,024	93.7%	(89.2-96.6%)	58.9%	0.012	87.3%	(80.1-92.4%)	62.3%	0.018		
CEB Broth	1	1	67	96.2%	(86.8-99.5%)	—	—	85.7%	(57.2-98.2%)	—	—		
BPW + Bolton	1	2	240	97.0%	(92.6-99.0%)	0.0%	0.000	88.6%	(73.3-96.2%)	0.0%	0.000		

By Food Matrix

Subgroup	Comparisons	Samples	Sensitivity	95% CI	I^2	Specificity	95% CI	I^2	Q-between	p-value
Chicken/Poultry	20	1,189	95.4%	(92.5-97.4%)	68.2%	88.7%	(83.4-92.7%)	65.4%	6.4	0.17
Carcass Rinse	8	1,032	96.8%	(93.7-98.6%)	52.1%	92.3%	(85.2-96.4%)	48.7%		
Multiple Matrices	10	2,560	97.8%	(96.4-98.8%)	45.3%	91.8%	(87.9-94.7%)	52.6%		
Turkey/Environmental	4	266	94.3%	(88.5-97.6%)	0.0%	80.4%	(67.2-89.6%)	0.0%		
Pork/Mixed	1	50	83.5%	(62.7-94.8%)	—	50.0%	(18.7-81.3%)	—		

By Sample Size Category

Subgroup	Comparisons	Mean Sample Size	Sensitivity	95% CI	I^2	Specificity	95% CI	I^2	Q-between	p-value
Small (<50)	10	27	91.8%	(86.2-95.6%)	54.2%	77.3%	(66.8-85.4%)	48.9%	12.3	0.006
Medium (50-200)	23	98	96.4%	(93.9-98.0%)	62.7%	91.5%	(87.4-94.6%)	58.3%		
Large (>200)	10	346	97.6%	(95.8-98.8%)	48.1%	92.8%	(88.7-95.7%)	52.4%		
